# Genomic classification of intrapulmonary metastasis and multiple primary lung cancer

**DOI:** 10.1002/ctm2.70463

**Published:** 2025-08-27

**Authors:** Jeongsoo Won, Yeon Seung Chung, Se‐Young Jo, Jiho Park, Hyo Sup Shim, Sangwoo Kim

**Affiliations:** ^1^ Department of Biomedical Systems Informatics Graduate School of Medical Science, Brain Korea 21 Project, Yonsei University College of Medicine Seoul South Korea; ^2^ Department of Pathology, Severance Hospital Yonsei University College of Medicine Seoul South Korea; ^3^ Department of Pathology Green Cross Laboratories Gyeonggi South Korea; ^4^ Department of Pathology Eone Laboratories Gangnam Seoul South Korea

## Graphical abstract

•MeTel is a novel Bayesian algorithm that accurately classifies multifocal lung cancers.

•It outperforms previous methods and is robust across different sequencing platforms.

•Its clinical utility was validated by correctly reclassifying diagnostically challenging cases.

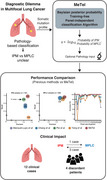

1

Dear Editor,

The most critical decision point in the diagnosis of multiple lung cancers is distinguishing between intrapulmonary metastasis (IPM) and multiple primary lung cancer (MPLC), as this differentiation significantly affects staging, treatment, and prognosis.^1^, ^2^ With growing emphasis on molecular profiling, the latest guidelines highlight its role in distinguishing IPM from MPLC, aiming to overcome the limitations of classic histological criteria.^3^, ^4^ However, both conventional histology‐based^5^, ^6^ and recently proposed genomic approaches^7^, ^8^ have shown limited reliability in clinical decision‐making. In this study, we developed MeTel (Metastasis Teller), a probabilistic Bayesian model that robustly classifies multifocal lung cancers as either IPM or MPLC. MeTel is designed for non‐small cell lung cancer (NSCLC), which represents the majority of multifocal lung cancer cases and for which somatic mutation data are widely available through routine clinical sequencing. Unlike previous approaches, MeTel does not rely on empirically defined thresholds or data‐driven approaches, thereby enhancing generalisability across sequencing platforms and patient populations.

The overall workflow of MeTel evaluates the profile of somatic mutations from different tumour sites (Figures 1 and S1). Tumours are first evaluated for key driver mutations (Table S1); if discordant, MPLC is immediately assigned. For concordant cases, MeTel calculates the probabilities of IPM and MPLC classification, providing two outputs: a classification score and a confidence level (‘Confident’ or ‘Likely’). For ‘Likely’ cases, defined by low variant counts, MeTel allows for post‐adjustment using Comprehensive Histological Assessment (CHA).^6^ Detailed descriptions of the MeTel algorithm are provided in the Supplementary Methods.

**FIGURE 1:**
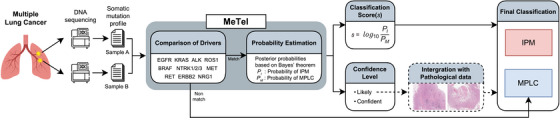
Overview of the MeTel algorithm, MeTel takes in the input somatic mutation profile with variant allele frequency (VAF) from the DNA sequencing data of multiple lung cancer samples as input. First, MeTel compares the driver mutations (EGFR, KRAS, ALK, ROS1, BRAF, NTRK1/2/3, MET, RET, ERBB2, and NRG1). If there are different drivers, they are classified as MPLC, and if the drivers match, the algorithm proceeds to further steps. MeTel estimates the probability of IPM (PI) and MPLC (PM) and outputs a classification score (*s*), which is the log‐scale value of the ratio of PI to PM. If *s* > 0, the classification is IPM; otherwise, it is MPLC. MeTel also provides a confidence level of either ‘Likely’ or ‘Confident’, based on the maximum number of mutations in the two tumours. ‘Likely’ is assigned when both tumours have two or fewer mutations; otherwise, the confidence level is ‘Confident’. When the confidence level is ‘Likely’, MeTel suggests an optional process that integrates histopathology data and uses the result for the final classification.

We compared MeTel with four previously described methods using independent test datasets (Table S2). Of the 635 samples in the test set, MeTel accurately classified 623 cases, with an error rate of 1.89%, outperforming all four other methods (Figure 2A, Table S3, and Note S1). Moreover, MeTel achieved the highest Cohen *κ* score of.95 (Figure 2B and Table S3), indicating a high inter‐rater reliability. The *F*1 scores for IPM (.97) and MPLC (.99) were also the best for MeTel (Figure 2C and Table S4). Notably, MeTel maintained high accuracy across the entire range of gene panel sizes (93.75%–100% for 4–808 genes and whole‐exome sequencing (WES)), whereas the four previous algorithms showed inconsistent accuracy across different panel sizes and platforms (Figure 2D). This performance stability indicates the strength of MeTel's probabilistic model, which avoids fixed cutoffs and training dependencies. Its robustness across sequencing platforms and panel sizes supports MeTel's applicability in diverse clinical settings.

**FIGURE 2:**
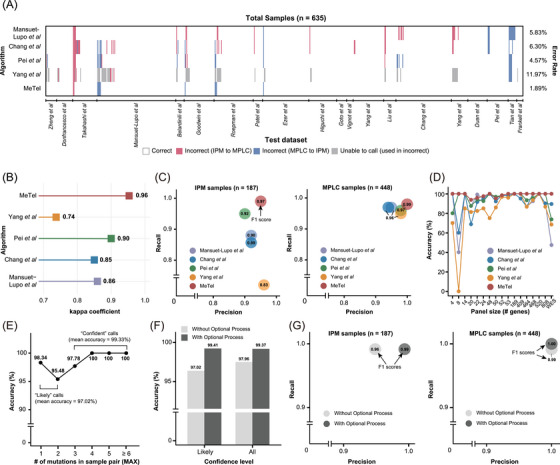
Performance of algorithms, (A) Classification results of the algorithms for test dataset (*n* = 635). The values on the right indicate the error rates for each algorithm across the entire test dataset. (B) The Cohen *κ* scores of the algorithms. (C) Precision and Recall for IPM and MPLC samples, respectively. The values inside the circles represent the *F*1 scores. (D) The accuracy of the algorithms for the dataset using different panel sizes. (E) Accuracy by the maximum mutation count of the two tumours (confidence level: ‘Likely’ for ≤ 2, ‘Confident’ for ≥ 3). (F) Changed accuracy at ‘Likely’ and all samples underwent the optional process. (G) Precision and recall before and after the optional process.

The confidence level assigned by MeTel provided practical guidance in clinical decision‐making. We found that almost all miscalls (10/12) by MeTel were included in the 336 ‘Likely’ calls (accuracy = 97.02%), whereas the 299 ‘Confident’ calls were 99.33% accurate (2/12 miscalls) (Figure 2E). As low confidence arises from limited genomic data, MeTel enables the integration of histological information for ‘Likely’ calls. When this optional process was applied, the overall accuracy of MeTel was increased to 99.37% (Figure 2F), with *F*1 scores of.99 and 1.00 for IPM and MPLC, respectively (Figure 2G). This demonstrates that MeTel provides highly accurate classifications based solely on mutation profiles and can achieve near‐perfect accuracy when supplemented with histologic information. Moreover, this result shows the algorithm's flexibility, enabling integrative diagnosis that combines mutation‐based classification with pathological assessments, particularly in ambiguous cases.

We further applied MeTel to 12 in‐house patients with multifocal NSCLC from Yonsei University Severance Hospital in Seoul, Korea (Tables S5 and S6), whose initial classifications were equivocal under standard procedures by pathologists, as described in the Supplementary Methods (section: In‐House Cohort for Clinical Application). We reviewed the 12 cases with additional follow‐up information, including disease progression, metastasis, and sequencing data (Table S7). In eight cases, MeTel's classifications aligned with the initial histopathologic diagnoses and were further supported by prognostic and follow‐up data (Figures S2 and S3). In contrast, four cases (Patients 2, 6, 7 and 11) exhibited discordance between histologic diagnosis and MeTel, and underwent in‐depth reassessment. Patient 2 had three adenocarcinomas with acinar‐predominant morphology. Although the second and third tumours exhibited micropapillary and lepidic features suggestive of MPLC, MeTel predicted IPM. This prediction was subsequently confirmed by CT imaging and WES‐based clonality analysis, leading to a revised diagnosis (Figure 3A and Note S2). Patient 6, originally diagnosed with IPM due to similar morphology of two squamous cell carcinomas, was reclassified as MPLC based on MeTel's prediction. This was supported by distinct clonal architectures (Figure 3B and Note S3). Patient 7 had two lepidic‐predominant adenocarcinomas separated by 8 years and was initially diagnosed as MPLC. However, MeTel predicted IPM, which was substantiated by copy number variation (CNV) similarity and clinical course (Figure 3C, Table S8 and Note S4). Patient 11 presented with two histologically dissimilar adenocarcinomas diagnosed 1 year apart. Although originally diagnosed as MPLC, MeTel predicted IPM, supported by shared rare variants and similar CNV patterns (Figure 3D, Table S9 and Note S5). These cases show the advantage of using genomic information in distinguishing IPM from MPLC, particularly when the clinical diagnosis is uncertain. They not only validate MeTel's utility in guiding reassessment but also reveal the limitations of conventional criteria – such as time interval or histologic similarity – in accurately classifying multifocal lung cancers. Supporting discussion is provided in Note S6.

**FIGURE 3:**
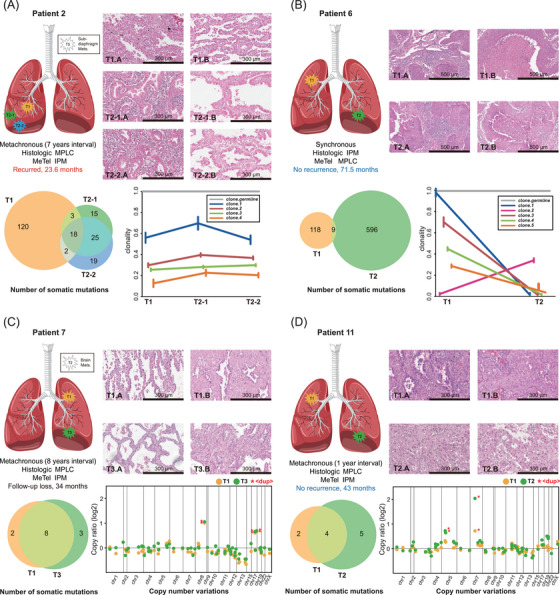
Discordant cases between the histologic predictions and MeTel analysis for the in‐house dataset. T refers to the tumour, with the number that follows indicating the order of occurrence. NGS analysis was performed for Patients 2 and 6, above using whole exome sequencing (WES), and for Patients 7 and 11 below, using the TSO500 panel sequencing. The microscopic slides display the representative histological patterns of each tumour. Venn diagrams represent the counts of the shared and unique somatic mutations. The line graphs show the results of the clonality analysis, depicting the clones constituting each tumour. In the case of panel sequencing data, the copy number variant (CNV) results were included, and the red asterisk (*) indicates duplication. (A) Patient 2: T1.A: acinar‐predominant component, T1.B: lepidic component, T2‐1A: acinar‐predominant component, T2‐1B: lepidic component. T2‐2A: acinar‐predominant component, and T2‐2B: lepidic component. There were 18 mutations shared by all tumours, and 120, 15 and 19 unique mutations were discovered in the three tumours, which exhibited similar clonal composition. (B) Patient 6: T1.A: inflammatory stroma, T1.B: necrosis, T2.A: inflammatory stroma and T2.B: necrosis. They shared 9 mutations and had 118 and 596 unique mutations. In addition, the profiles of the clones constituting each tumour were different. (C) Patient 7: T1.A: lepidic‐predominant component, T1.B: acinar component, T3.A: lepidic‐predominant component, and T3.B: acinar component. The tumours shared 8 mutations and had 2 and 3 unique mutations, respectively. Furthermore, they had three copy number duplications in the same positions. (D) Patient 11: T1.A: acinar predominant component, T1.B: solid component, T2.A: solid predominant component, and T2.B: complex glandular component. They shared 4 mutations and had 2 and 5 unique mutations. They showed the same copy number duplication in chromosome 7.

In conclusion, MeTel provides a statistically principled, training‐free, and platform‐independent approach for distinguishing IPM from MPLC using somatic mutation data. Its Bayesian framework computes posterior probabilities based on the number and frequency of shared variants, without relying on arbitrary cutoffs or large labelled datasets, thereby ensuring consistent performance across sequencing platforms and patient populations. In cases with low variant counts – and therefore lower‐confidence predictions – MeTel also allows the optional integration of histopathologic information to support clinical decision‐making. The robustness of the current model provides a strong foundation for future enhancements. Our preliminary explorations into CNV integration and ethnicity‐specific modelling confirm the feasibility of these extensions and underscore the critical need for larger genomic databases to further advance the field (Notes S7 and S8). Ultimately, applying MeTel to larger prospective cohorts will be essential to further establish its clinical utility.

## AUTHOR CONTRIBUTIONS

**Jeongsoo Won**: conceptualisation, methodology, software, validation, formal analysis, investigation, data curation, writing – original draft, visualisation. **Yeon Seung Chung**: conceptualisation, validation, formal analysis, investigation, writing – original draft, visualisation. **Se‐Young Jo**: methodology, software, formal analysis. **Jiho Park**: methodology, software, formal analysis. **Hyo Sup Shim**: conceptualisation, resources, writing – review and editing, supervision, project administration. **Sangwoo Kim**: conceptualisation, methodology, formal analysis, resources, writing – original draft, writing – review and editing, supervision, project administration.

## CONFLICT OF INTEREST STATEMENT

S.K. is a cofounder of AIMA Inc., which seeks to develop techniques for early cancer diagnosis based on circulating tumour DNA.

## FUNDING

This study was supported by the Bio&Medical Technology Development Program of the National Research Foundation (NRF) funded by the Korean government (MSIT) (RS‐2023‐00261820) to S.K. and the National Research Foundation of Korea (NRF) grant funded by the Korean Government (MSIT) (RS‐2025‐00518452) to H.S.S.

## ETHICS STATEMENT

This study involving human participants was reviewed and approved by the Ethics and Human Subjects Committee of Severance Hospital, Korea (Approval No. 4‐2017‐1149).

## Supporting information



Supporting Information

Supporting Information

Supporting Information

## Data Availability

The raw sequencing in‐house data generated from this study are available under NCBI Sequence Read Archive (SRA) (SRA code: PRJNA1070282), which can be accessed at https://www.ncbi.nlm.nih.gov/bioproject/PRJNA1070282. The source codes for MeTel algorithm are available at https://github.com/JeongsooWon/MeTel.
