# Supplementary material for: Genomic classification of intrapulmonary metastasis and multiple primary lung cancer
Source: Clin Transl Med. 2025 Aug 27;15(9):e70463. doi: 10.1002/ctm2.70463 (PMC12390766; doi:10.1002/ctm2.70463)
Supplement: Supplementary file 1 — Supporting Information [file CTM2-15-e70463-s001.pdf]

Table S1. Clinically Actionable Driver Alterations Used for Initial Discordance Filtering in MeTel

Molecular alteration	Description
EGFR exon 19 deletion or exon 21 L858R mutation	Positive
EGFR S768I, L861Q, and/or G719X mutation	Positive
EGFR exon 20 insertion	Positive
KRAS G12C mutation	Positive
ALK rearrangement	Positive
ROS1 rearrangement	Positive
BRAF V600E mutation	Positive
NTRK1/2/3 fusion	Positive
MET exon 14 skipping mutation	Positive
RET rearrangement	Positive
ERBB2 (HER2) mutation	Positive
NRG1 gene fusion	Positive

List of clinically actionable driver alterations used in the MeTel's initial discordance-based classification, based on the NCCN Clinical Practice Guidelines for Oncology (Version 6.2025) for Non-Small Cell Lung Cancer. The presence of discordant driver alterations between tumor samples leads to immediate classification as multiple primary lung cancer (MPLC).

Table S2. Summary of test dataset

Study	Number of pairs			Clinical information available			Panel size (number of covered genes)
	IPM	MPLC	Total	Histologic pattern	Time interval	AIS/MIA/Lepidic	
Zheng et al	4	10	14	Available (14)	NA	NA	4, 48
Donfrancesco et al	6	14	20	Available (20)	NA	Available (20)	14
Takahashi et al	20	12	32	NA	Available (32)	NA	20
Mansuet-Lupo et al	33	76	109	Discordant cases (30)	Discordant cases (30)	Discordant cases (30)	22
Belardinili et al	1	9	10	Available (10)	Available (10)	Available (10)	22
Goodwin et al	7	33	40	Available (40)	Available (40)	Available (40)	24
Roepman et al	12	41	53	Available (53)	Available (53)	Available (53)	50
Patel et al	3	8	11	Available (11)	Available (11)	Available (11)	50
Ezer et al	9	54	63	NA	NA	NA	52
Higuchi et al	8	29	37	Available (37)	Available (37)	Available (37)	53
Goto et al	0	12	12	Available (12)	Available (12)	Available (12)	53
Vignot et al	7	0	7	Primary tumor only (7)	Available (7)	NA	189
Yang et al	22	19	41	Available (41)	Discordant cases (6)	Discordant cases (6)	409
Liu et al	3	12	15	Available (15)	NA	Available (15)	464
Chang et al	25	51	76	NA	Available (76)	NA	468
Yang et al	20	1	21	NA	Available (21)	NA	8, 468
Duan et al	3	22	25	Available (25)	Available (25)	Available (25)	520
Pei et al	4	26	30	NA	NA	NA	808
Tian et al	0	11	11	Available (11)	Synchronous only (10)	Available (11)	WES
Frankell et al	0	8	8	Available (8)	Available (8)	Available (8)	WES

IPM, intrapulmonary metastasis; MPLC, multiple primary lung cancers; AIS, adenocarcinoma in situ; MIA, minimally invasive adenocarcinoma; WES, whole exome sequencing; NA, not available

Total 20 datasets: 635 pairs (187 IPM / 448 MPLC).

Table S3. Performance of algorithms (635 cases from 20 test datasets)

		**Mansuet-Lupo *et al.***		**Chang *et al.***		**Pei *et al.***		**Yang *et al.***			**MeTel**	
		IPM	MPLC	IPM	MPLC	IPM	MPLC	IPM	MPLC	Inconclusive	IPM	MPLC
**Final Classification**	IPM	164	23	161	26	178	10	135	8	44	185	2
	MPLC	14	434	14	434	19	428	5	424	19	10	438
**Error Rate (Model development dataset excluded)**	**Kappa coefficient**	5.83% (7.03)	0.86	6.30% (7.16)	0.85	4.57% (4.79)	0.90	11.97% (12.79)	0.74		1.89%	0.96

IPM, intrapulmonary metastasis; MPLC, multiple primary lung cancer.

Table S4. F1 scores for IPM and MPLC algorithms (635 cases from 20 test datasets)

	**Mansuet-Lupo *et al.***		**Chang *et al.***		**Pei *et al.***		**Yang *et al.***		**MeTel**	
	IPM	MPLC	IPM	MPLC	IPM	MPLC	IPM	MPLC	IPM	MPLC
**Precision**	0.92	0.95	0.92	0.94	0.90	0.98	0.96	0.98	0.95	1.00
**Recall**	0.88	0.97	0.86	0.97	0.95	0.96	0.72	0.95	0.99	0.98
**F1 score**	0.90	0.96	0.89	0.96	0.92	0.97	0.83	0.96	0.97	0.99

IPM, intrapulmonary metastasis; MPLC, multiple primary lung cancer.

Table S5. Patient characteristics of the targeted DNA sequencing (TSO500) and WES datasets

Patient characteristics (n = 12)	Targeted sequencing (n = 6)	WES (n = 6)
Sex, n (%)		
Male	2 (33.3)	2 (33.3)
Female	4 (66.7)	4 (66.7)
Median age at first resection, y (range)	64.5 (45-74)	65 (56-72)
Smoking status, n (%)		
Current/Ex-smoker	0 (0.0)	2 (33.3)
Nonsmoker	6 (100.0)	4 (66.7)
Median pack-year of smoker (range)	-	27.5 (25-30)
Synchronicity, n (%)		
Synchronous	2 (33.3)	3 (50.0)
Metachronous	4 (66.7)	3 (50.0)
Median time interval between resections in metachronous case, m (range)	63.7 (13.3-129.8)	63.4 (60.8-75.8)
Distribution of tumors, n (%)		
Ipsilateral (same lobe)	1 (16.7)	2 (33.3)
Ipsilateral (different lobe)	3 (50.0)	1 (16.7)
Contralateral	2 (33.3)	3 (50.0)

TSO500, TruSight Oncology 500; WES, whole exome sequencing.

Table S6 Clinical and pathological characteristics of 25 multiple lung cancer tumors

Patient	Tumor ID	Sex	Age (at first resection)	Predominant histologic pattern	Minor histologic pattern	Lepidic component	CHA	MeTel	Seq format	Location	Time interval (m)	Size (cm)	High Tumor grade	IASLC grade	Lymphovascular invasion	Pleural invasion	N stage	Smoking status	P-Y	DFS (m)	Recur	OS (m)	Death
1) Recurrent tumor with a long-time interval																							
Pt 1	T1	F	56	A complex glandular	A acinar	N	IPM	IPM	WES	LUL	60.8	3	Y	3	N	N	2	nonsmoker		0.6	Y	44.9	N
	T2			A complex glandular	A acinar	N				RUL		1	Y	3	N	Y							
Pt 2	T1	F	71	A lepidic	A lepidic	Y	MPLC	IPM	WES	RML	75.8	1.7	N	1	N	N	0	nonsmoker		23.6	Y	66.8	N
	T2-1			A acinar	A papillary	Y				RLL		1.7	N	2	N	N							
	T2-2			A acinar	A lepidic	Y				RLL		1.2	N	2	N	N							
Pt 5	T1	F	68	A papillary	A acinar	N	IPM	IPM	WES	RUL	63.4	2.7	N	2	N	N	0	nonsmoker		21.4	N	21.4	N
	T2			A papillary	A acinar	N				LUL		1.2	N	2	N	N							
Pt 7	T1	M	66	A lepidic	A acinar	Y	MPLC	IPM	TSO500	LUL	99.3	3	N	1	N	N	0	nonsmoker		34.1	NA	34.1	NA
	T3			A lepidic	A acinar	Y				LLL		2	N	1	N	N	0						
Pt 8	T1	F	45	A micropapillary	A papillary	N	IPM	IPM	TSO500	LUL	129.8	2	Y	3	Y	N	0	nonsmoker		32.9	N	32.9	N
	T2			A micropapillary	A papillary	N				LLL		1	Y	3	N	N	0						
2) Synchronous multiple squamous cell carcinoma																							
Pt 6	T1	M	64	Squamous	Squamous	NA	IPM	MPLC	WES	LLL	0.1	2.5	-	-	N	N	0	exsmoker	30	71.5	N	71.5	N
	T2			Squamous	Squamous	NA				RUL		2.5	-	-	Y	N							
3) Ambiguous histology with identical driver gene status																							
Pt 3	T1	F	59	A acinar	A papillary	Y	IPM	IPM	WES	LLL	0	0.9	N	2	N	N	0	nonsmoker		24.1	N	24.1	N
	T2			A acinar	A papillary	N				LLL		1.3	N	2	N	N							
Pt 4	T1	M	66	A papillary	A acinar + A micropapillary	Y	IPM	IPM	WES	LLL	0	2	Y	2	N	N	0	current	25	31.4	N	31.4	N
	T2			A papillary	A acinar	N				LLL		1.5	N	2	N	N							
Pt 9	T1	M	63	A acinar	A papillary + A micropapillary	N	IPM	IPM	TSO500	LUL	5.3	1.2	Y	3	N	N	0	nonsmoker		32.1	N	32.1	N
	T2			A acinar	A micropapillary + A papillary	N				LUL		0.7	Y	3	N	N	0						
Pt 10	T1	F	55	A papillary	A acinar	N	IPM	IPM	TSO500	LUL	28.1	2.8	N	2	N	N	0	nonsmoker		66.2	N	66.2	N
	T2			A papillary	A acinar+ A micropapillary	N				LLL		1.3	Y	3	N	Y	0						
Pt 11	T1	F	74	A acinar	A solid	N	MPLC	IPM	TSO500	RUL	13.3	2.9	Y	3	N	N	0	nonsmoker		43	N	43	N
	T2			A solid	A acinar	N				LLL		2.2	Y	3	N	N	0						
Pt 12	T1	F	67	A acinar	A papillary	Y	MPLC	MPLC	TSO500	LUL	0.5	1.8	N	2	N	N	2	nonsmoker		9.9	Y	67.3	Y
	T2			A papillary	A micropapillary	Y				RML		1.9	Y	2	N	N	2						

CHA, comprehensive histologic assessment; P-Y, pack-year; DFS, disease-free survival; OS, overall survival; Pt, patient; T, tumor; F, female; M, male; A, adenocarcinoma; IPM, intrapulmonary metastasis; MPLC, multiple primary lung cancer; WES, whole exome sequencing; LUL, left upper lobe; LLL, left lower lobe; RUL, right upper lobe; RML, right middle lobe; RLL, right lower lobe; NA, not available

High tumor grade : Mucinous, solid, cribriform, micropapillary

For Non-mucinous adenocarcinoma. 1 = well, 2 = intermediate, 3 = poor

Table S7. Results applied to in-house data

Patient	Tumor	Histology (predominant)	Lepidic	TI (m)	Initial Dx	M-L et al.*	Chang et al.*	Pei et al.	Yang et al.	MeTel	Re-Adj Dx
Pt 1	T1	A acinar	N	60.8	IPM	MPLC	IPM	IPM	IPM	IPM	IPM
	T2	A acinar	N								
Pt 2	T1	A lepidic	Y	75.8	MPLC	MPLC	MPLC	IPM	IPM	IPM	IPM
	T2-1	A acinar	Y								
	T2-2	A acinar	Y								
Pt 3	T1	A acinar	Y	0.0	IPM	IPM	IPM	IPM	IPM	IPM	IPM
	T2	A acinar	N								
Pt 4	T1	A papillary	Y	0.0	IPM	IPM	IPM	IPM	IPM	IPM	IPM
	T2	A papillary	N								
Pt 5	T1	A papillary	N	63.4	IPM	MPLC	IPM	IPM	IPM	IPM	IPM
	T2	A papillary	N								
Pt 6	T1	Squamous	NA	0.1	IPM	IPM	IPM	IPM	IPM	MPLC	MPLC
	T2	Squamous	NA								
Pt 7	T1	A lepidic	Y	99.3	MPLC	MPLC	MPLC	IPM	IPM	IPM	IPM
	T3	A lepidic	Y								
Pt 8	T1	A micropapillary	N	129.8	IPM	MPLC	IPM	IPM	IPM	IPM	IPM
	T2	A micropapillary	N								
Pt 9	T1	A acinar	N	5.3	IPM	IPM	IPM	IPM	IPM	IPM	IPM
	T2	A acinar	N								
Pt 10	T1	A papillary	N	28.1	IPM	IPM	IPM	IPM	IPM	IPM	IPM
	T2	A papillary	N								
Pt 11	T1	A acinar	N	13.3	MPLC	IPM	IPM	IPM	IPM	IPM	IPM
	T2	A solid	N								
Pt 12	T1	A acinar	Y	0.5	MPLC	MPLC	MPLC	MPLC	MPLC	MPLC	MPLC
	T2	A papillary	Y								

Pt, patient; T, tumor; A, adenocarcinoma; TI, time interval; Dx, diagnosis; M-L, Mansuet-Lupo; Re-Adj, re-adjudicated; IPM, intrapulmonary metastasis; MPLC, multiple primary lung cancers; NA, not available.
*, algorithm including histological classification.

Table S8. CNV results (Pt7)

#CHROM	POS	REF	Tumor	ALT	INFO	FORMAT	FC	Tumor	ALT	INFO	FORMAT	FC
chr1	40356095	C		.	END=40372764;ANT=MYCL1	FC	0.967		.	END=40372764;ANT=MYCL1	FC	1.129
chr1	115245084	T		.	END=115261621;ANT=NRAS	FC	1.005		.	END=115261621;ANT=NRAS	FC	0.941
chr1	204485505	C		.	END=204526342;ANT=MDM4	FC	1.171		.	END=204526342;ANT=MDM4	FC	1.295
chr2	16075982	C		.	END=16090656;ANT=MYCN	FC	0.81		.	END=16090656;ANT=MYCN	FC	1.059
chr2	29416088	G		.	END=30143527;ANT=ALK	FC	0.939		.	END=30143527;ANT=ALK	FC	0.999
chr3	12626011	A		.	END=12704516;ANT=RAF1	FC	0.988		.	END=12704516;ANT=RAF1	FC	0.99
chr3	138374229	C		.	END=138478187;ANT=PIK3CB	FC	1.025		.	END=138478187;ANT=PIK3CB	FC	0.958
chr3	178866308	T		.	END=178952154;ANT=PIK3CA	FC	1.055		.	END=178952154;ANT=PIK3CA	FC	1.005
chr3	195776752	A		.	END=195806640;ANT=TFRC	FC	1.036		.	END=195806640;ANT=TFRC	FC	1.04
chr4	1793038	C		.	END=1812559;ANT=FGFR3	FC	0.809		.	END=1812559;ANT=FGFR3	FC	0.943
chr4	55106263	G		.	END=55163711;ANT=PDGFRA	FC	1.001		.	END=55163711;ANT=PDGFRA	FC	1.076
chr4	55524180	C		.	END=55604995;ANT=KIT	FC	0.905		.	END=55604995;ANT=KIT	FC	1.02
chr4	81186341	C		.	END=81211595;ANT=FGF5	FC	0.918		.	END=81211595;ANT=FGF5	FC	0.97
chr4	123747929	G		.	END=123817823;ANT=FGF2	FC	1.052		.	END=123817823;ANT=FGF2	FC	1.027
chr5	38942404	G		.	END=39074481;ANT=RICTOR	FC	1.137		.	END=39074481;ANT=RICTOR	FC	0.933
chr5	44305095	C		.	END=44388786;ANT=FGF10	FC	0.957		.	END=44388786;ANT=FGF10	FC	1.031
chr5	141974853	C		.	END=142076962;ANT=FGF1	FC	1.072		.	END=142076962;ANT=FGF1	FC	1.253
chr5	149493399	G		.	END=149530880;ANT=PDGFRB	FC	1.016		.	END=149530880;ANT=PDGFRB	FC	1.258
chr5	176512220	C		.	END=176527047;ANT=FGFR4	FC	1.117		.	END=176527047;ANT=FGFR4	FC	1.331
chr6	41903676	G		.	END=42014927;ANT=CCND3	FC	0.987		.	END=42014927;ANT=CCND3	FC	0.988
chr6	152021630	C		.	END=152420103;ANT=ESR1	FC	0.997		.	END=152420103;ANT=ESR1	FC	1.073
chr7	55086969	C		.	END=55273312;ANT=EGFR	FC	0.979		.	END=55273312;ANT=EGFR	FC	0.981
chr7	92243233	G		.	END=92462639;ANT=CDK6	FC	0.997		.	END=92462639;ANT=CDK6	FC	0.977
chr7	116339137	T		.	END=116436180;ANT=MET	FC	0.812		.	END=116436180;ANT=MET	FC	0.911
chr7	140434395	T		.	END=140624505;ANT=BRAF	FC	0.973		.	END=140624505;ANT=BRAF	FC	0.946
chr8	31497499	C		.	END=32621922;ANT=NRG1	FC	1.018		.	END=32621922;ANT=NRG1	FC	1.251
chr8	38271144	A		.	END=38326324;ANT=FGFR1	FC	0.998		.	END=38326324;ANT=FGFR1	FC	1.191
chr8	128743613	A		<DUP>	SVTYPE=CNV;END=128758574;ANT=MYC	FC	1.955		<DUP>	SVTYPE=CNV;END=128758574;ANT=MYC	FC	2.205
chr9	4994244	A		.	END=5126793;ANT=JAK2	FC	1.012		.	END=5126793;ANT=JAK2	FC	0.997
chr10	43572705	C	T1	.	END=43623989;ANT=RET	FC	0.863	T3	.	END=43623989;ANT=RET	FC	0.997
chr10	89624225	A		.	END=89726498;ANT=PTEN	FC	1.056		.	END=89726498;ANT=PTEN	FC	0.916
chr10	103529086	G		.	END=103542197;ANT=FGF8	FC	0.847		.	END=103542197;ANT=FGF8	FC	0.974
chr10	123239093	T		.	END=123353603;ANT=FGFR2	FC	0.929		.	END=123353603;ANT=FGFR2	FC	0.95
chr11	69456080	C		.	END=69471087;ANT=CCND1	FC	0.904		.	END=69471087;ANT=CCND1	FC	0.981
chr11	69510405	T		.	END=69523684;ANT=FGF19	FC	0.877		.	END=69523684;ANT=FGF19	FC	0.926
chr11	69583096	G		.	END=69594022;ANT=FGF4	FC	0.865		.	END=69594022;ANT=FGF4	FC	1.066
chr11	69623035	T		.	END=69635441;ANT=FGF3	FC	0.873		.	END=69635441;ANT=FGF3	FC	1.048
chr11	108098350	C		.	END=108236237;ANT=ATM	FC	1.051		.	END=108236237;ANT=ATM	FC	1.017
chr11	125496029	C		.	END=125545482;ANT=CHEK1	FC	0.964		.	END=125545482;ANT=CHEK1	FC	1.052
chr12	4476292	C		.	END=4490788;ANT=FGF23	FC	0.9		.	END=4490788;ANT=FGF23	FC	1.03
chr12	4539807	A		.	END=4559806;ANT=FGF6	FC	0.84		.	END=4559806;ANT=FGF6	FC	0.981
chr12	25359177	A		.	END=25401358;ANT=KRAS	FC	1.002		.	END=25401358;ANT=KRAS	FC	0.909
chr12	56474083	T		.	END=56497311;ANT=ERBB3	FC	0.798		.	END=56497311;ANT=ERBB3	FC	0.832
chr12	58136509	C		.	END=58150859;ANT=CDK4	FC	0.879		.	END=58150859;ANT=CDK4	FC	0.967
chr12	69202256	G		.	END=69238168;ANT=MDM2	FC	0.968		.	END=69238168;ANT=MDM2	FC	0.894
chr13	22245512	A		.	END=22278213;ANT=FGF9	FC	0.733		.	END=22278213;ANT=FGF9	FC	0.722
chr13	32890596	A		.	END=32972909;ANT=BRCA2	FC	0.844		.	END=32972909;ANT=BRCA2	FC	0.744
chr13	102375179	G		.	END=103054030;ANT=FGF14	FC	0.731		.	END=103054030;ANT=FGF14	FC	0.737
chr13	113951748	C		.	END=113977608;ANT=LAMP1	FC	0.731		.	END=113977608;ANT=LAMP1	FC	0.694
chr15	49716493	T		.	END=49762320;ANT=FGF7	FC	1.129		.	END=49762320;ANT=FGF7	FC	1
chr17	37844989	C		<DUP>	SVTYPE=CNV;END=37884569;ANT=ERBB2	FC	1.579		<DUP>	SVTYPE=CNV;END=37884569;ANT=ERBB2	FC	1.68
chr17	41197309	G		.	END=41276383;ANT=BRCA1	FC	0.935		.	END=41276383;ANT=BRCA1	FC	1.042
chr17	57970543	C		<DUP>	SVTYPE=CNV;END=58027235;ANT=RPS6KB1	FC	1.616		<DUP>	SVTYPE=CNV;END=58027235;ANT=RPS6KB1	FC	1.747
chr19	30300900	T		.	END=30314953;ANT=CCNE1	FC	0.991		.	END=30314953;ANT=CCNE1	FC	1.054
chr19	40736221	C		.	END=40789214;ANT=AKT2	FC	1.01		.	END=40789214;ANT=AKT2	FC	1.01
chr19	45853248	C		.	END=45875268;ANT=ERCC2	FC	0.919		.	END=45875268;ANT=ERCC2	FC	0.998
chr19	45908590	T		.	END=45927472;ANT=ERCC1	FC	0.875		.	END=45927472;ANT=ERCC1	FC	0.946
chr22	29083883	G		.	END=29137479;ANT=CHEK2	FC	1.003		.	END=29137479;ANT=CHEK2	FC	0.898
chrX	66764987	G		.	END=66943685;ANT=AR	FC	0.963		.	END=66943685;ANT=AR	FC	0.947

Table S9. CNV results (Pt11)

#CHROM	POS	REF	Tumor	ALT	INFO	FORMAT	FC	Tumor	ALT	INFO	FORMAT	FC
chr1	40356095	C		.	END=40372764;ANT=MYCL1	FC	0.984		.	END=40372764;ANT=MYCL1	FC	1.024
chr1	115245084	T		.	END=115261621;ANT=NRAS	FC	1.041		.	END=115261621;ANT=NRAS	FC	0.926
chr1	204485505	C		.	END=204526342;ANT=MDM4	FC	0.958		.	END=204526342;ANT=MDM4	FC	0.921
chr2	16075982	C		.	END=16090656;ANT=MYCN	FC	1.058		.	END=16090656;ANT=MYCN	FC	1.06
chr2	29416088	G		.	END=30143527;ANT=ALK	FC	1.037		.	END=30143527;ANT=ALK	FC	1.182
chr3	12626011	A		.	END=12704516;ANT=RAF1	FC	0.927		.	END=12704516;ANT=RAF1	FC	0.948
chr3	138374229	C		.	END=138478187;ANT=PIK3CB	FC	0.912		.	END=138478187;ANT=PIK3CB	FC	0.9
chr3	178866308	T		.	END=178952154;ANT=PIK3CA	FC	0.916		.	END=178952154;ANT=PIK3CA	FC	0.962
chr3	195776752	A		.	END=195806640;ANT=TFRC	FC	0.921		.	END=195806640;ANT=TFRC	FC	0.885
chr4	1793038	C		.	END=1812559;ANT=FGFR3	FC	0.923		.	END=1812559;ANT=FGFR3	FC	0.797
chr4	55106263	G		.	END=55163711;ANT=PDGFRA	FC	1.069		.	END=55163711;ANT=PDGFRA	FC	1.117
chr4	55524180	C		.	END=55604995;ANT=KIT	FC	0.989		.	END=55604995;ANT=KIT	FC	1.02
chr4	81186341	C		.	END=81211595;ANT=FGF5	FC	0.96		.	END=81211595;ANT=FGF5	FC	1.097
chr4	123747929	G		.	END=123817823;ANT=FGF2	FC	1.035		.	END=123817823;ANT=FGF2	FC	1.166
chr5	38942404	G		.	END=39074481;ANT=RICTOR	FC	1.257		<DUP>	SVTYPE=CNV;END=39074481;ANT=RICTOR	FC	1.618
chr5	44305095	C		.	END=44388786;ANT=FGF10	FC	1.212		<DUP>	SVTYPE=CNV;END=44388786;ANT=FGF10	FC	1.534
chr5	141974853	C		.	END=142076962;ANT=FGF1	FC	1.03		.	END=142076962;ANT=FGF1	FC	1.201
chr5	149493399	G		.	END=149530880;ANT=PDGFRB	FC	1.027		.	END=149530880;ANT=PDGFRB	FC	1.16
chr5	176512220	C		.	END=176527047;ANT=FGFR4	FC	1.107		.	END=176527047;ANT=FGFR4	FC	1.132
chr6	41903676	G		.	END=42014927;ANT=CCND3	FC	0.97		.	END=42014927;ANT=CCND3	FC	0.988
chr6	152021630	C		.	END=152420103;ANT=ESR1	FC	0.95		.	END=152420103;ANT=ESR1	FC	0.937
chr7	55086968	G		<DUP>	SVTYPE=CNV;END=55273312;ANT=EGFR	FC	1.661		<DUP>	SVTYPE=CNV;END=55273312;ANT=EGFR	FC	4.016
chr7	92243233	G		.	END=92462639;ANT=CDK6	FC	1.122		.	END=92462639;ANT=CDK6	FC	1.159
chr7	116339137	T		.	END=116436180;ANT=MET	FC	1.094		.	END=116436180;ANT=MET	FC	1.229
chr7	140434395	T		.	END=140624505;ANT=BRAF	FC	1.104		.	END=140624505;ANT=BRAF	FC	1.164
chr8	31497499	C		.	END=32621922;ANT=NRG1	FC	0.905		.	END=32621922;ANT=NRG1	FC	0.936
chr8	38271144	A		.	END=38326324;ANT=FGFR1	FC	0.933		.	END=38326324;ANT=FGFR1	FC	0.895
chr8	128743614	T		.	END=128758574;ANT=MYC	FC	1.033		.	END=128758574;ANT=MYC	FC	0.981
chr9	4994244	A		.	END=5126793;ANT=JAK2	FC	0.89		.	END=5126793;ANT=JAK2	FC	0.976
chr10	43572705	C	T1	.	END=43623989;ANT=RET	FC	0.983	T2	.	END=43623989;ANT=RET	FC	0.985
chr10	89624225	A		.	END=89726498;ANT=PTEN	FC	0.879		.	END=89726498;ANT=PTEN	FC	0.993
chr10	103529086	G		.	END=103542197;ANT=FGF8	FC	0.897		.	END=103542197;ANT=FGF8	FC	0.891
chr10	123239093	T		.	END=123353603;ANT=FGFR2	FC	0.987		.	END=123353603;ANT=FGFR2	FC	0.928
chr11	69456080	C		.	END=69471087;ANT=CCND1	FC	1.076		.	END=69471087;ANT=CCND1	FC	1.07
chr11	69510405	T		.	END=69523684;ANT=FGF19	FC	0.982		.	END=69523684;ANT=FGF19	FC	0.988
chr11	69583096	G		.	END=69594022;ANT=FGF4	FC	1.13		.	END=69594022;ANT=FGF4	FC	1.08
chr11	69623035	T		.	END=69635441;ANT=FGF3	FC	1.177		.	END=69635441;ANT=FGF3	FC	1.099
chr11	108098350	C		.	END=108236237;ANT=ATM	FC	1.025		.	END=108236237;ANT=ATM	FC	1.065
chr11	125496029	C		.	END=125545482;ANT=CHEK1	FC	1.052		.	END=125545482;ANT=CHEK1	FC	1.012
chr12	4476292	C		.	END=4490788;ANT=FGF23	FC	1.036		.	END=4490788;ANT=FGF23	FC	0.923
chr12	4539807	A		.	END=4559806;ANT=FGF6	FC	1.069		.	END=4559806;ANT=FGF6	FC	0.95
chr12	25359177	A		.	END=25401358;ANT=KRAS	FC	0.873		.	END=25401358;ANT=KRAS	FC	0.879
chr12	56474083	T		.	END=56497311;ANT=ERBB3	FC	0.916		.	END=56497311;ANT=ERBB3	FC	0.911
chr12	58136509	C		.	END=58150859;ANT=CDK4	FC	0.96		.	END=58150859;ANT=CDK4	FC	0.994
chr12	69202256	G		.	END=69238168;ANT=MDM2	FC	0.949		.	END=69238168;ANT=MDM2	FC	0.99
chr13	22245512	A		.	END=22278213;ANT=FGF9	FC	0.91		.	END=22278213;ANT=FGF9	FC	0.81
chr13	32890596	A		.	END=32972909;ANT=BRCA2	FC	0.931		.	END=32972909;ANT=BRCA2	FC	0.961
chr13	102375179	G		.	END=103054030;ANT=FGF14	FC	0.892		.	END=103054030;ANT=FGF14	FC	0.758
chr13	113951748	C		.	END=113977608;ANT=LAMP1	FC	0.942		.	END=113977608;ANT=LAMP1	FC	0.801
chr15	49716493	T		.	END=49762320;ANT=FGF7	FC	1.087		.	END=49762320;ANT=FGF7	FC	0.966
chr17	37844990	A		.	END=37884569;ANT=ERBB2	FC	1.114		.	END=37884569;ANT=ERBB2	FC	1.251
chr17	41197309	G		.	END=41276383;ANT=BRCA1	FC	1.106		.	END=41276383;ANT=BRCA1	FC	1.092
chr17	57970544	C		.	END=58027235;ANT=RPS6KB1	FC	1.058		.	END=58027235;ANT=RPS6KB1	FC	1.093
chr19	30300900	T		.	END=30314953;ANT=CCNE1	FC	1.053		.	END=30314953;ANT=CCNE1	FC	1.392
chr19	40736221	C		.	END=40789214;ANT=AKT2	FC	1.091		.	END=40789214;ANT=AKT2	FC	1.376
chr19	45853248	C		.	END=45875268;ANT=ERCC2	FC	1.058		.	END=45875268;ANT=ERCC2	FC	1.374
chr19	45908590	T		.	END=45927472;ANT=ERCC1	FC	0.957		.	END=45927472;ANT=ERCC1	FC	1.304
chr22	29083883	G		.	END=29137479;ANT=CHEK2	FC	1.017		.	END=29137479;ANT=CHEK2	FC	0.857
chrX	66764987	G		.	END=66943685;ANT=AR	FC	1.235		.	END=66943685;ANT=AR	FC	1.115

Table S10. Classification Performance of MeTel Across Different Values of Mutation Frequency Parameter (*f_v*)

**Default f_v**	**Accuracy**	**Precision (IPM / MPLC)**	**Recall (IPM / MPLC)**	**F1-score (IPM / MPLC)**
10^{-5}	98.11%	0.95 / 1.00	0.99 / 0.98	0.97 / 0.99
10^{-6}	98.11%	0.95 / 1.00	0.99 / 0.98	0.97 / 0.99
10^{-7}	98.11%	0.95 / 1.00	0.99 / 0.98	0.97 / 0.99

Performance metrics (accuracy, precision, recall, and F1-score) for MeTel classification were evaluated under three different values of the mutation frequency parameter (f_v): 10^{-5}, 10^{-6}, and 10^{-7}. All metrics remained identical across the three settings, indicating that model performance is robust to changes in.

Table S11. Classification results with and without VAF imputation

case	Classification Score(s)	Diagnosis Result	Confidence Level	Classification_Score VAF03(s)	Diagnosis_Result VAF03	Confidence_Level VAF03
P1	58.32348	IPM	Confident	36.71582	IPM	Confident
P2 (T1-T2.1)	73.76374	IPM	Confident	40.7273	IPM	Confident
P2 (T1-T2.2)	73.72242	IPM	Confident	40.7273	IPM	Confident
P2 (T2.1-T2.2)	202.329	IPM	Confident	213.2118	IPM	Confident
P3	41.29899	IPM	Confident	40.66313	IPM	Confident
P4	142.7949	IPM	Confident	143.2851	IPM	Confident
P5	68.80765	IPM	Confident	61.80208	IPM	Confident
P6	-28.1324	MPLC	Confident	-198.499	MPLC	Confident
P7	45.21707	IPM	Confident	45.04047	IPM	Confident
P8	4.280739	IPM	Confident	2.205771	IPM	Confident
P9	33.28815	IPM	Confident	33.88211	IPM	Confident
P10	5.397491	IPM	Confident	4.991351	IPM	Confident
P11	19.15582	IPM	Confident	19.80024	IPM	Confident
P12	-1.89801	MPLC	Confident	-3.17444	MPLC	Confident

MeTel classification results in the in-house cohort using original VAF values and with all VAFs replaced by 0.3. The diagnosis and confidence levels remained consistent.

Table S12. Histology–genomic concordance across included test datasets

Study	Number of pairs (based on genomics)			Number of histology-genomic concordant pairs			Panel size (number of covered genes)
	IPM	MPLC	Total	IPM	MPLC	Total	
Zheng et al	4	10	14	4	1	5	4, 48
Donfrancesco et al	6	14	20	4	10	14	14
Takahashi et al	20	12	32	6	5	11	20
Mansuet-Lupo et al	33	76	109	29	50	79	22
Belardinili et al	1	9	10	0	8	8	22
Goodwin et al	7	33	40	6	18	24	24
Roepman et al	12	41	53	8	27	35	50
Patel et al	3	8	11	2	7	9	50
Ezer et al	9	54	63	6	46	52	52
Higuchi et al	8	29	37	4	27	31	53
Goto et al	0	12	12	0	10	10	53
Vignot et al	7	0	7	7	0	7	189
Yang et al	22	19	41	13	13	26	409
Liu et al	3	12	15	1	12	13	464
Chang et al	25	51	76	13	45	58	468
Yang et al	20	1	21	0	0	0	8, 468
Duan et al	3	22	25	3	4	7	520
Pei et al	4	26	30	0	0	0	808
Tian et al	0	11	11	0	7	7	WES
Frankell et al	0	8	8	0	7	7	WES
Total	187	448	635	106	297	403	

Summary of test cases with concordant histologic and genomic classification across 20 studies. This subset (n = 403) was used for sensitivity analysis under stricter ground-truth assumptions.

Table S13. Classification results with and without CNV integration

case	Classification Score(s)	Diagnosis	Confidence_Level	Classification Score CNV	Diagnosis CNV	Confidence_Level CNV
P1	58.3235	IPM	Confident	59.9686	IPM	Confident
P2 (T1-T2.1)	73.7637	IPM	Confident	80.3135	IPM	Confident
P2 (T1-T2.2)	73.7224	IPM	Confident	79.5602	IPM	Confident
P2 (T2.1-T2.2)	202.329	IPM	Confident	195.862	IPM	Confident
P3	41.299	IPM	Confident	40.5878	IPM	Confident
P4	142.795	IPM	Confident	141.474	IPM	Confident
P5	68.8076	IPM	Confident	63.6312	IPM	Confident
P6	-28.1324	MPLC	Confident	-21.2528	MPLC	Confident

Summary of MeTel classification scores and outputs for eight in-house tumor pairs, comparing the original model and the CNV-integrated version. All cases remained correctly classified.
