# Supplementary material for: Genomic classification of intrapulmonary metastasis and multiple primary lung cancer
Source: Clin Transl Med. 2025 Aug 27;15(9):e70463. doi: 10.1002/ctm2.70463 (PMC12390766; doi:10.1002/ctm2.70463)
Supplement: Supplementary file 2 — Supporting Information [file CTM2-15-e70463-s003.pdf]

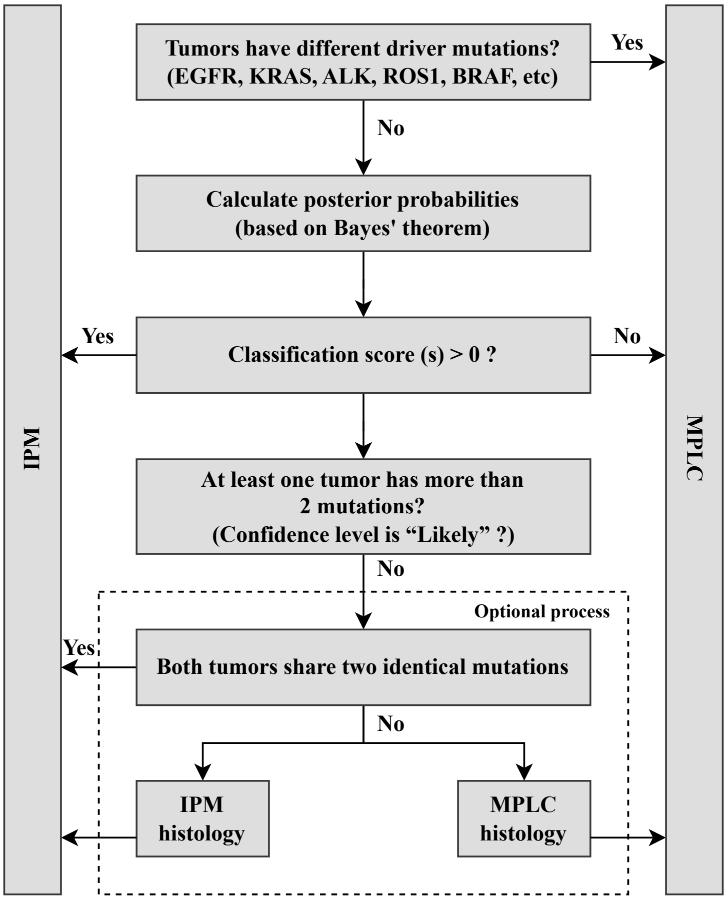

Figure S1. Overview of the MeTel algorithm.

MeTel compares driver mutations (EGFR, KRAS, ALK, ROS1, BRAF, NTRK1/2/3, MET, RET, ERBB2, and NRG1). If there are different drivers, they are classified as MPLC, and if the drivers match, MeTel proceeds to estimate the probability of IPM (P_I) and MPLC (P_M) and outputs a classification score (s), which is the log-scale value of the ratio of P_I to P_M. If s is greater than 0, the classification is IPM; otherwise, it is MPLC.

Cases with two or fewer total mutations across both tumors are assigned a confidence level of “Likely”. Within these “Likely” cases, if both mutations are identical between the two tumors (i.e., two shared somatic mutations), the case is classified as IPM due to the improbability of such a coincidence. In all other “Likely” cases, the final classification is determined based on comprehensive histological assessment (CHA), which evaluates major and minor histologic patterns, as well as cytologic and architectural features.

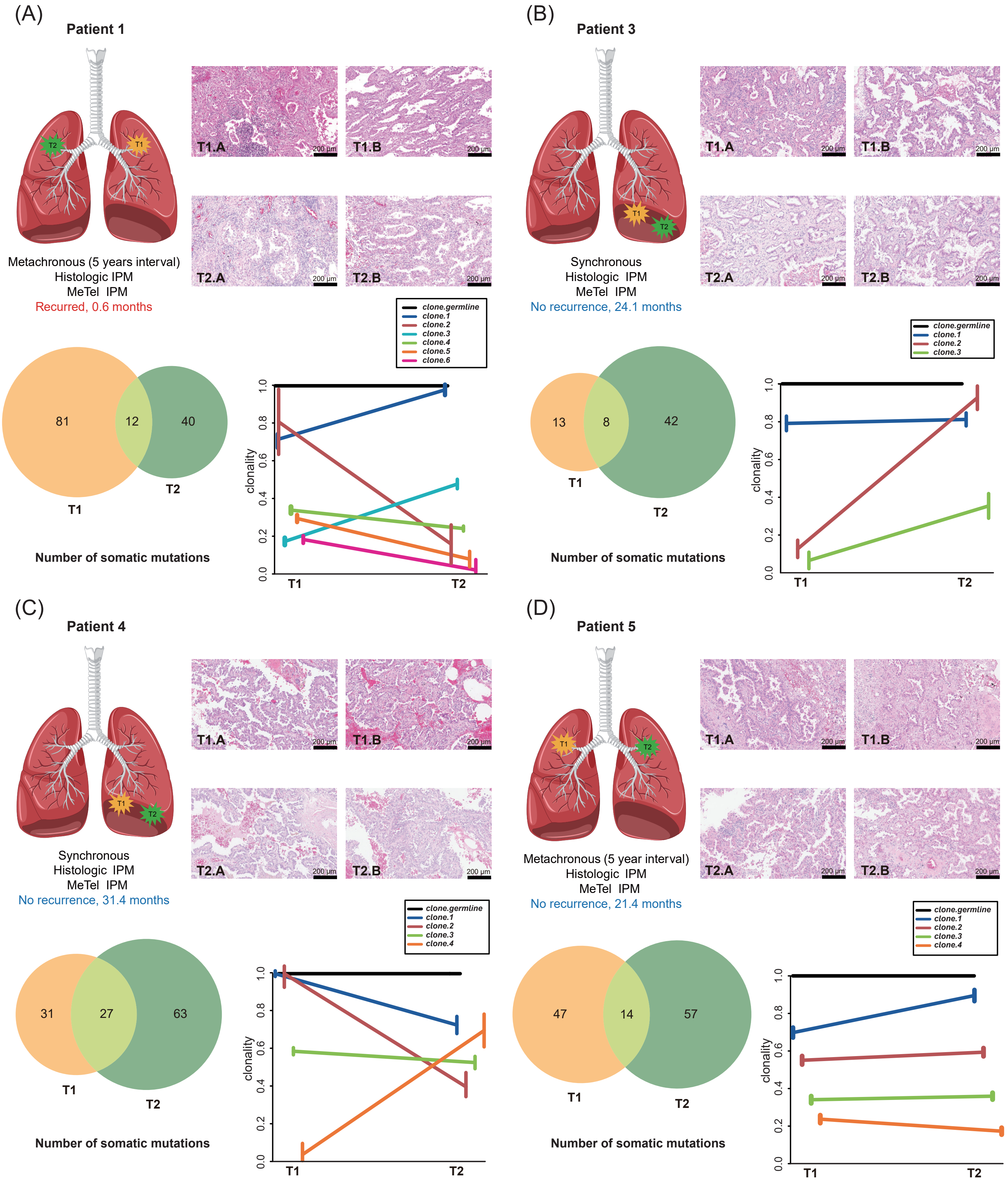

Figure S2. Cases consistent between histologic predictions and MeTel analysis of the in-house dataset (WES). Histologic images of the primary (T1) and secondary (T2) tumors in patients and results of genomic analysis (num-ber of shared mutations and clonal composition). (A) Patient 1: Tumors were separated by a 5-year interval (H&E stain). Both tumors showed a predominantly complex glandular pattern with a minor acinar pattern. Histology and MeTel analyses interpreted as intrapulmonary metastasis. (B) Patient 3: Two synchronous tumors. Both tumors showed a predominantly acinar pattern with a minor papillary pattern. Histology and MeTel analyses interpreted as intrapulmonary metastasis. (C) Patient 4: Two synchronous tumors. Both tumors showed a predominantly papillary pattern with a minor acinar pattern. Histology and MeTel analyses interpreted as intrapulmonary metastasis. (D) Tumors in Patient 5 were separated by a 5-year interval. Both tumors showed a predominantly papillary pattern with a minor acinar pattern. Histology and MeTel analyses interpreted as intrapulmonary metastasis.

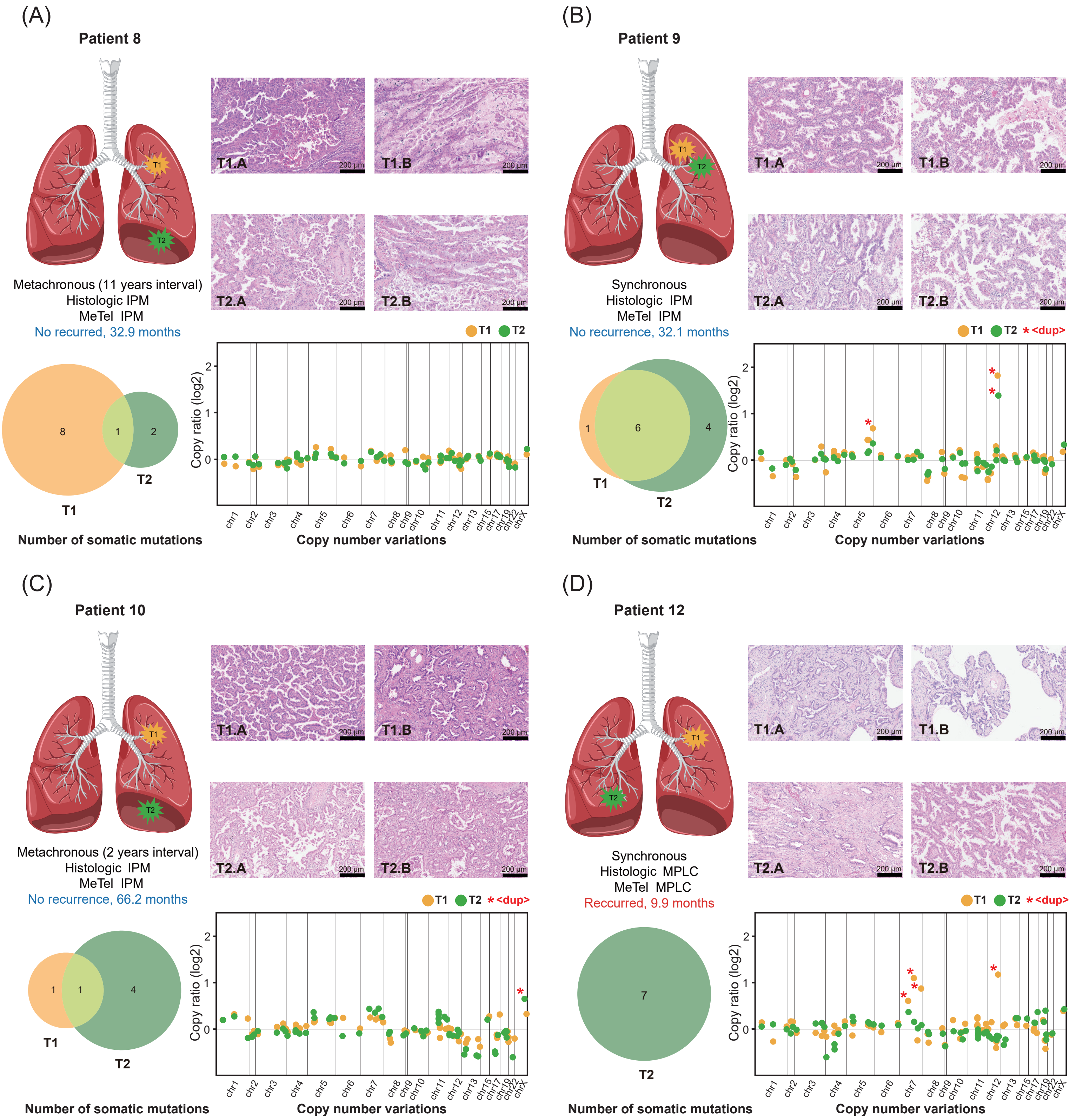

Figure S3. Cases consistent between histologic predictions and MeTel analysis of the in-house dataset (TSO500 panel sequencing). Histologic images of the primary (T1) and secondary (T2) tumors in patients and results of genomic analysis (number of shared mutations and CNV pattern). (A) Patient 8 were separated by an 11-year interval (H&E stain). Both tumors predominantly exhibit a micropapillary pattern and a characteristic mucinous component, indicating lung adenocarcinoma harboring an ALK rearrangement. Both tumors showed ALK immunopositivity, and histology and MeTel analyses interpreted them as intrapulmonary metastasis. (B) Patient 9 were separated by synchronous tumors. Both tumors predominantly showed a acinar pattern with minor papillary pattern. Histology and MeTel analyses interpreted as intrapulmo-nary metastasis. (C) Patient 10 were separated by a two-year interval. Both tumors predominantly showed a papillary pattern with minor acinar pattern. Histology and MeTel analyses interpreted as intrapulmonary metastasis. (D) Two synchronous tumors in Patient 12. Both tumors shared similar histologic patterns (acinar and papillary) but differ in the proportion of these components, with acinar predominance in T1 and papillary predominance in T2. No somatic mutations were observed in T1. Histology and MeTel analyses interpreted them as multiple primary lung cancers.

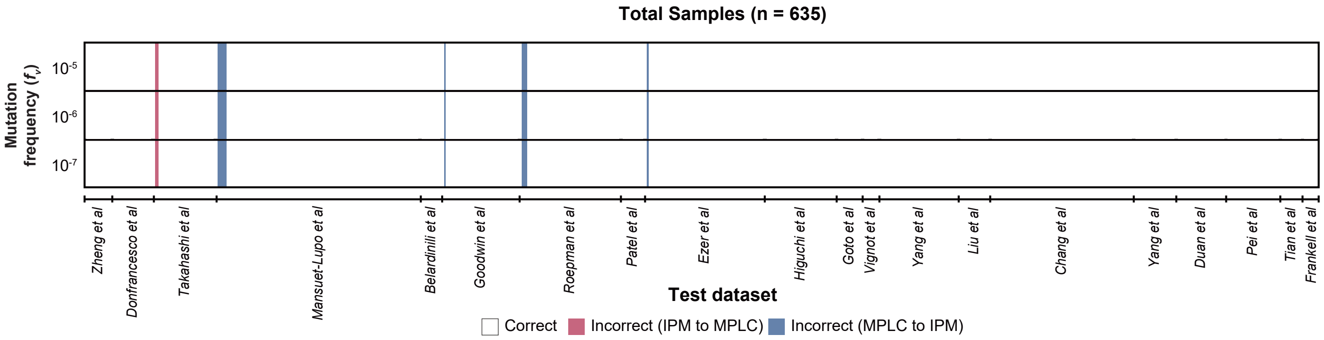

Figure S4. Sensitivity analysis of MeTel's classification outcomes across different values of the background mutation frequency parameter (f_V)

Classification outcomes across different values of the background mutation frequency parameter (f_V). The results were identical for all three values tested (10^{-5}, 10^{-6}, and 10^{-7}).

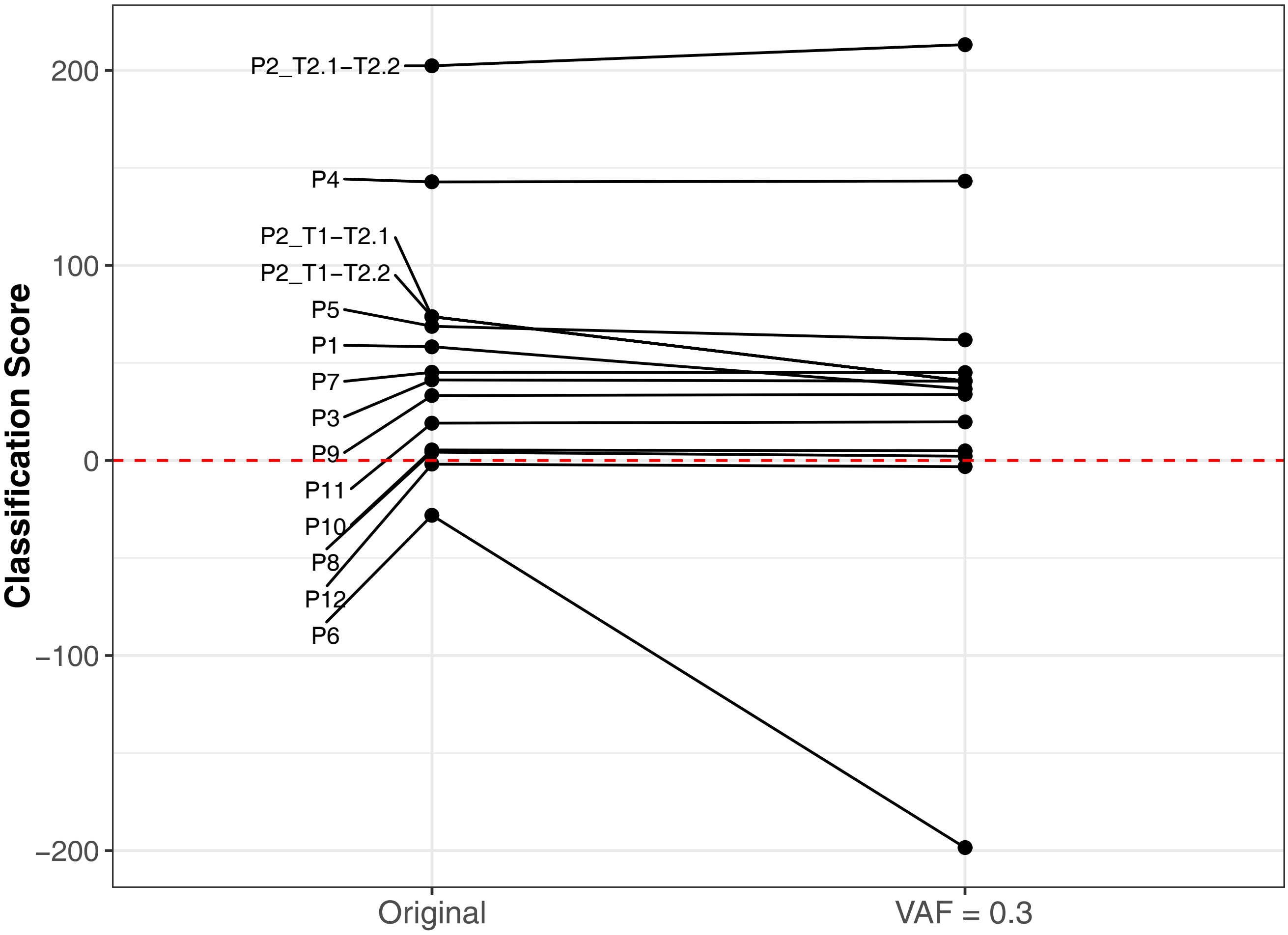

Figure S5. Sensitivity analysis for VAF imputation

Comparison of classification scores before and after replacing all VAFs with 0.3 in the in-house cohort. No changes were observed in diagnosis results.

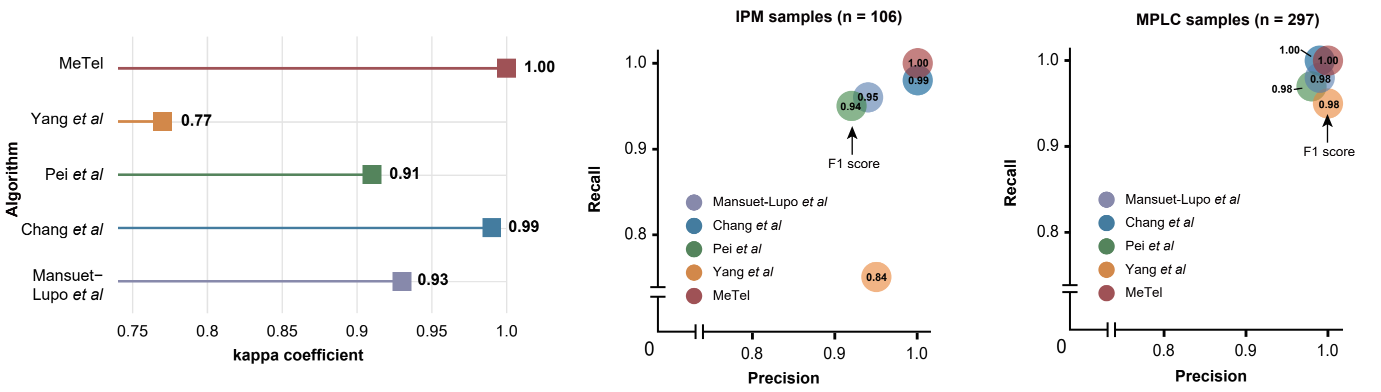

Figure S6. Performance comparison in histology–genomic concordant cases

Classification performance of MeTel and four previous algorithms evaluated in the concordant subset. MeTel achieved perfect agreement (kappa = 1.00) and F1 score of 1.00 for both IPM (n = 106) and MPLC (n = 297), confirming its robustness even under stringent validation.

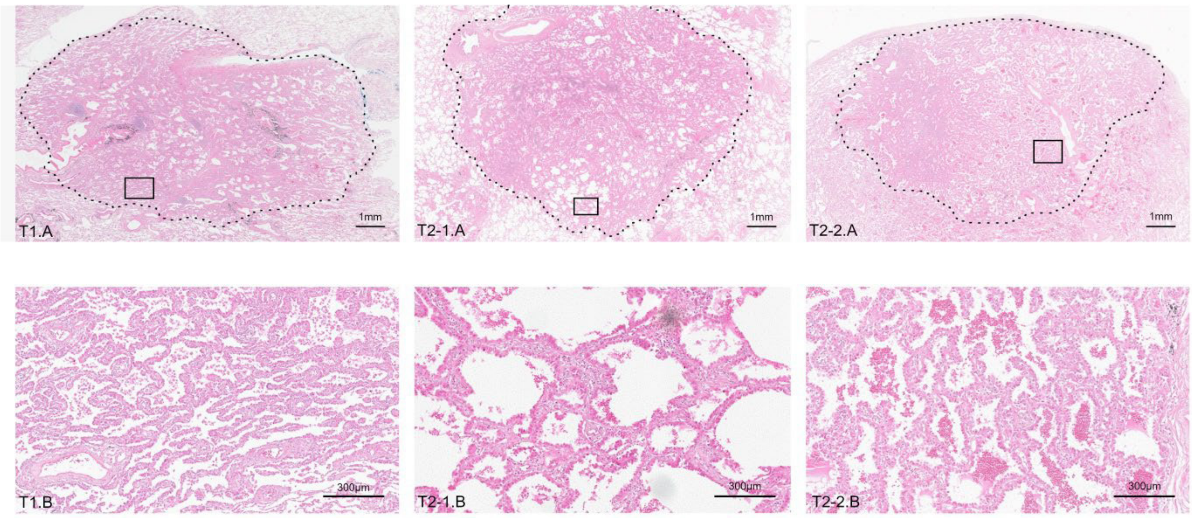

Figure S7. Pathologic slides of three tumors (T1, T2-1, and T2-2) of Patient 2
Overview of each tumor with the black box highlighting the lepidic area (T1.B). Higher magnification of the box in the early tumor displays an iatrogenic collapse pattern, which is interpreted as a non-invasive component (T2-1.B and T2-2.B). Higher magnification of the box in subsequent tumors shows extensive epithelial proliferation, indicative of an invasive outgrowth pattern.

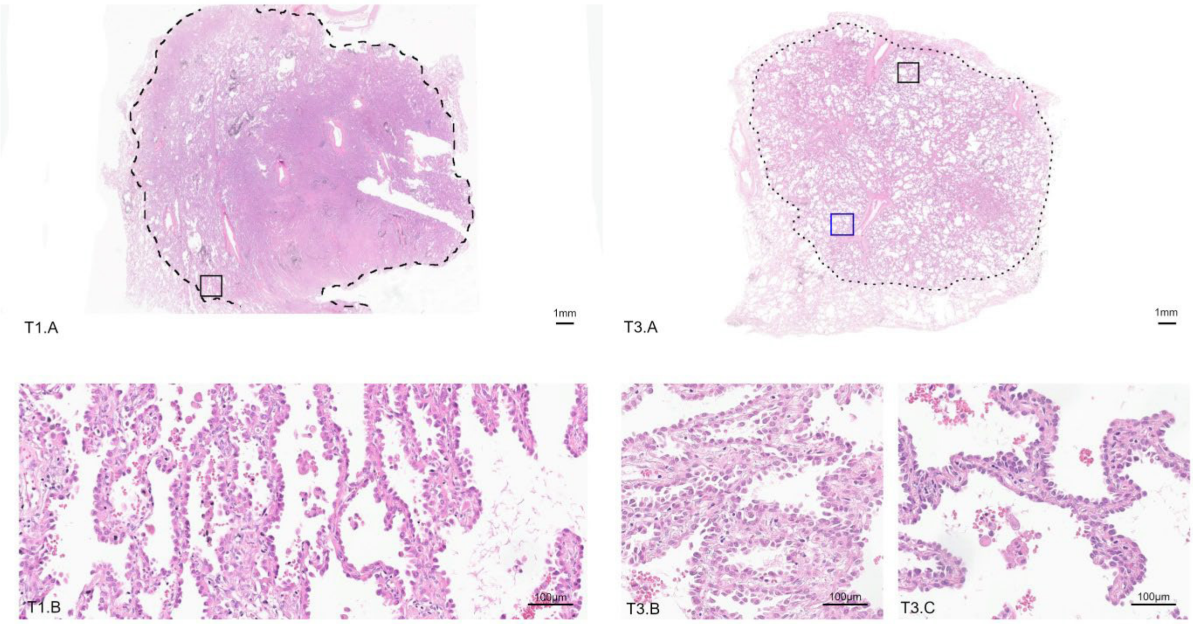

Figure S8. Pathological slides of two intrapulmonary tumors (T1 and T3) of Patient 7.
Overview of each tumor, with boxes highlighting the lepidic area (T1.B). Higher magnification of the box in the early tumor displays an iatrogenic collapse pattern, which is interpreted as a non-invasive component (T3.B). Higher magnification of the blue box in the subsequent tumor shows extensive epithelial proliferation (T3.C). Higher magnification of the black box shows definitive lepidic pattern, suggesting it was an equivocal case.

$$\frac{total\_cn}{major\_cn} \times VAF = \alpha$$

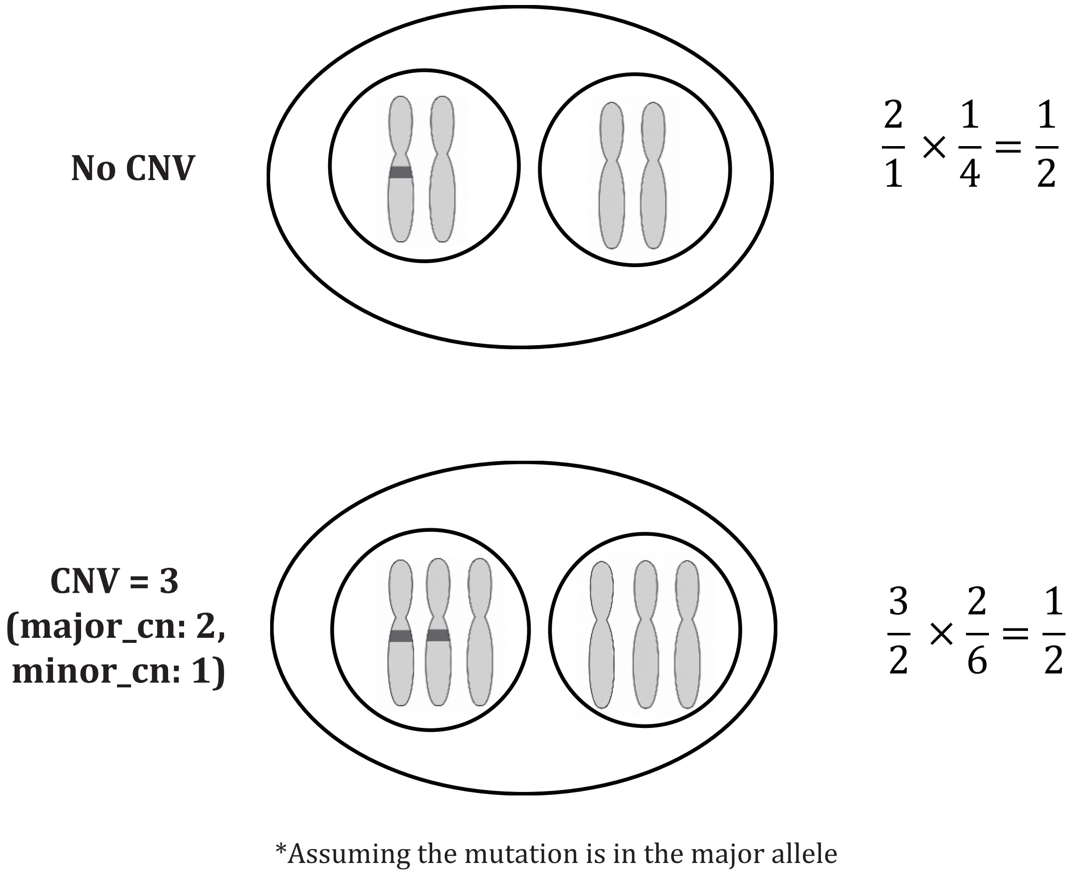

Figure S9. Conceptual model for CNV-integrated classification.
Schematic comparison of MeTel's original formulation (top) and the CNV-integrated version (bottom). The CNV-integrated approach adjusts classification by incorporating allele-specific copy number, assuming the mutation resides on the major allele.

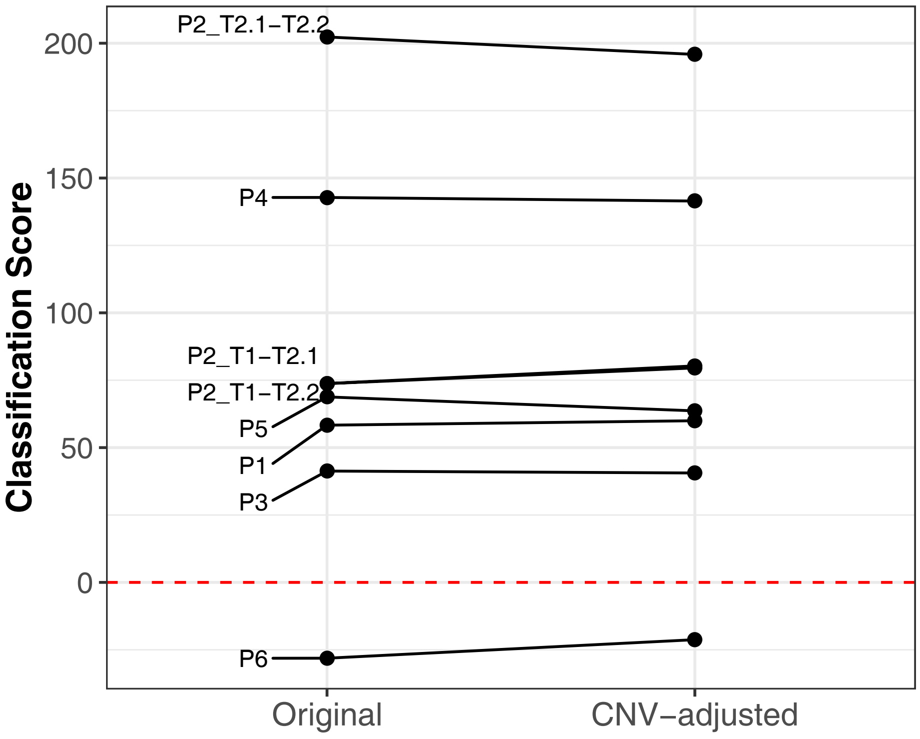

Figure S10. Comparison of MeTel classification scores with and without CNV integration

Classification scores before and after incorportating CNV information across eight in-house tumor pairs. All cases retained their original diagnostic labels.
