# Supplementary material for: Genomic classification of intrapulmonary metastasis and multiple primary lung cancer
Source: Clin Transl Med. 2025 Aug 27;15(9):e70463. doi: 10.1002/ctm2.70463 (PMC12390766; doi:10.1002/ctm2.70463)
Supplement: Supplementary file 3 — Supporting Information [file CTM2-15-e70463-s002.pdf]

Supplementary Methods

MeTel algorithm

MeTel uses the profile of somatic mutations and optionally their VAFs from tumors at two different sites from the same patient. At the time of data collection, MeTel scans are performed for the presence of key driver mutations (EGFR, KRAS, ALK, ROS1, BRAF, NTRK1/2/3, MET, RET, ERBB2, and NRG1) **(TableS1)** [1] in each sample. Discordance in the key driver mutations between the input tumors instantly leads to a decision of MPLC, thereby bypassing all other steps. Otherwise, MeTel proceeds to the following steps.

MeTel uses a training-free probabilistic model to calculate and compare two probabilities: P_M, the probability that tumors T_1 and T_2 are MPLC, and P_I, the probability that either T_1 or T_2 is IPM of the other, given the input data $D = D_1 \cup D_2$, where D_i is a set of somatic mutations (v) in tumor T_i. Using Bayes' theorem, the posterior probabilities after observing D are defined as

$$P_M = P(M|D) = P(D|M)P(M) = \prod_{v \in D} P(v|M)P(M) \quad \cdots (1)$$

$$P_I = P(I|D) = P(D|I)P(I) = \prod_{v \in D} P(v|I)P(I) \quad \cdots (2)$$

We calculate the likelihood terms $P(v|M)$ and $P(v|I)$. In MPLC, $P(v|M)$ is the likelihood that one or both tumors independently exhibit mutation v, which can be estimated from the global frequency of v in NSCLC.

$$P(v|M) \;=\; \begin{cases} f_v^2 & \text{if } v \in D_1 \cap D_2 \\ f_v(1-f_v) & \text{otherwise} \end{cases}$$

where f_v is the number of NSCLC cases that reported the presence of v divided by the total number of NSCLC cases in a public database AACR (American Association for Cancer Research) Project GENIE (Genomics Evidence Neoplasia Information Exchange) (v.15) [2]. If v is not present in the database, a minimal number of 10^{-6} is used for f_v. This minimal frequency was selected to reflect the higher mutational burden typically observed in cancer compared to normal tissues, where the somatic mutation rate is estimated to be approximately $2.7*10^{-9}$ [3]. To assess the robustness of this mutation frequency, we conducted a sensitivity analysis by varying f_v across 10^{-5}, 10^{-6}, and 10^{-7}. As shown in **Table S10**, performance metrics including accuracy, precision, recall, and F1-score remained unchanged across all three values.

Furthermore, **Figure S4** shows that the predicted labels were entirely consistent for all samples, indicating that MeTel's classification is robust to the choice of f_v within this range.

For IPM, $P(v|I)$ is calculated from three different cases. Assuming T_2 is a metastasis of T_1 (the opposite case is also considered, see below), the cases are either (i) both T_1 and T_2 exhibit v, (ii) only T_1 exhibits v, or (iii) only T_2 exhibits v. For (i), two scenarios are considered: (i-a) v occurred in T_1 and transferred to T_2, or (i-b) v occurred in T_1 and was not transferred to T_2, but v occurred in T2 independently. For (ii), the only possible scenario is that v occurred in T_1, and it was neither transferred to nor occurred in T_2. Likewise, for (iii), the only possible scenario is that v did not occur in T_1, but it occurred independently occurred in T_2. Therefore, the overall likelihood is calculated as follows:

$$P(v|I, T_1 \to T_2) = \begin{cases} f_v(\alpha_v + f_v(1-\alpha_v)) & \text{if } v \in D_1 \cap D_2 \\ f_v(1-\alpha_v)(1-f_v) & \text{if } v \in D_1, v \notin D_2 \\ (1-f_v)f_v & \text{if } v \notin D_1, v \in D_2 \end{cases}$$

where α_v is the allele fraction of v in the primary tumor, which is used to estimate the probability of mutation transfer at the time of metastasis. Assuming no copy number variation (CNV) or loss of heterozygosity (LOH), α_v is set to two times of VAF to account for the cellular fraction in the tumor, or 0.3 (mean allelic fraction in all variants in the GENIE database) if VAF is not provided. The upper limit of α_v is 1 – ε, where ε is a very small number used to avoid divergence in the final value. The direction of metastasis was chosen based on a higher likelihood, unless otherwise specified.

$$P(v|I) = \max\{P(v|I, T_1 \to T_2), P(v|I, T_2 \to T_1)\}$$

The prior probabilities $P(M)$ and $P(I)$ were set to 0.71 and 0.29, respectively, based on the observed ratio of MPLC and IPM from 1446 cases reported in 21 independent studies[4-24]. Finally, we calculate the P_M and P_I, by applying the calculated likelihoods $P(D|M)$ and $P(D|I)$ and the prior probabilities $P(M)$ and $P(I)$ to equation (1) and equation (2).

After the estimation process, MeTel outputs two values: the classification score and confidence. The classification score (s) is calculated by the ratio of P_I and P_M on a logarithmic scale:

$$s = \log_{10} P_I / P_M$$

A provisional call of prediction is IPM if $s > 0$ or MPLC otherwise.

The confidence is annotated with the provisional call in at two levels: "Likely" and "Confident".

MeTel assigns “Likely” when the maximum number of mutations in either tumor is two or fewer, and “Confident” when the number of mutations exceeds two.

For “Likely” cases, MeTel allows post-adjustment based on histopathological features. If two identical somatic mutations are shared between tumors, the case is classified as IPM, as the probability of observing such shared mutations independently is highly improbable. In all other “Likely” cases, the final classification is determined based on the Comprehensive Histological Assessment (CHA), a widely accepted histologic algorithm proposed by Girard et al. [25], which evaluates major and minor histologic components, along with cytologic and architectural features.

Acquisition of the independent test dataset

Genome sequencing and the annotated clinical decisions of 635 multifocal tumor pairs (187 IPM and 448 MPLC) from 20 previous studies were acquired [4-6,8,9,11-17,23,24,26-31]. The range of sequenced genes was from 4 to 808, including whole exome sequencing (WES). The diagnoses of the tumor pairs were collected from each original study and used as the answer set. Patients with distant (e.g., cutaneous or CNS), lymph node metastases, and samples without observed somatic mutations were excluded. Somatic mutation profiles were obtained from the original article. Clinical information, including histological patterns, time intervals, and adenocarcinoma in situ (AIS)/minimally invasive adenocarcinoma (MIA) predominance, was collected based on the available data.

These 635 tumor pairs were used to comparatively evaluate the performance of MeTel against existing classification methods. This dataset was not used for training or parameter fitting, as MeTel is a Bayesian classifier that does not require model learning.

Performance evaluation

MeTel was run on the test dataset with the default settings. The VAFs were set to 0.3 for the dataset without VAF information (based on the average VAF of all mutations in the GENIE database[2], which is used in algorithm development). To assess the effect of this imputation, we conducted a sensitivity analysis using the in-house cohort with known VAF values. The classification results remained unchanged when all VAFs were replaced with the default value

of 0.3, indicating that the imputation had no impact on MeTel's performance. **(Figure S5 and Table S11)** For cases with “Likely” confidence, the optional process using histopathological information was applied. The performance measurements for accuracy, kappa coefficient, precision, recall, and F1 score were calculated with and without the optional process.

Four algorithms from previous studies (Mansuet-Lupo *et al.* [9], Chang *et al.* [14], Pei *et al.* [16], and Yang *et al.* [17]) were applied to the same test dataset to evaluate their performance, and the protocols described in the original studies were followed. Detailed protocols for applying these algorithms in the present study are provided in below.

- Mansuet-Lupo *et al.* [9], Tumor pairs that included AIS or MIA or with a time interval ≥5 years were considered MPLC. When inconclusive, the number of shared somatic mutations was used (MPLC: <1, IPM: ≥2). In the case of a single shared mutation, the mutation frequency was considered. If the shared mutation was known to be a frequent driver in *KRAS* (p.G12X) or *EGFR* (E19del or p.L858R), the final judgment followed histological classification. Otherwise, the pairs were referred to as IPM.
- Chang *et al.* [14], Tumors with distinct histology or with AIS/MIA/lepidic predominance were classified as MPLC. In contrast, tumors that showed a complete overlap in morphology were identified as IPM. If the historical comparison was equivocal, tumor pairs with two or more shared somatic mutations were called IPM. Tumors with different driver mutations or no shared mutations are referred to as MPLC. The original study stated that tumors with a single shared mutation are probable IPM, while the population frequency should be considered. Because the exact protocol for population frequency examination was not defined, we classified these cases as IPM.
- Pei *et al.* [16], Tumors with no mutation in common and different driver gene hotspot mutations (*EGFR*, *KRAS, BRAF, ERBB2, ALK, ROS1, MET*, or *RET*) were classified as MPLC. Tumors were classified as IPM if they shared a driver mutation that was not *EGFR* L858R or if they shared *EGFR* L858R along with additional mutations. When *EGFR* L858R was the only shared mutation, tumors were classified as MPLC.
- Yang *et al.* [17], Tumor pairs with different drivers were classified as MPLC. If two tumors showed identical drivers or both were wild-type, they were determined whether they had share other somatic mutations. Tumors with identical somatic mutations, aside from those involving driver oncogenes, were classified as IPM. If no shared

mutation was found, compares the *TP53* mutations between the tumors. Tumors with different *TP53* mutations were classified as MPLC. Tumors that could not be classified according to these criteria were regarded as "inconclusive."

In-House Cohort for Clinical Application

We collected and reviewed the medical charts of patients with multiple NSCLCs who underwent surgical resection at Yonsei University Severance Hospital in Seoul, Korea, from 2006 to 2020. During this period, a total of 433 patients had two or more NSCLC tumors resected. Candidate selection was based on three criteria: (1) Recurrence of NSCLC after a long interval, where the tumors were generally considered MPLC without the need for histologic analysis; (2) cases that are challenging to classify using classic histologic criteria; and (3) cases where the classification as MPLC or IPM significantly changes the pathologic stage (e.g., multiple tumors occurred in the bilateral lung). Moreover, we collected 25 NSCLC tumor samples from 12 patients, as follows: (1) 11 tumors from 5 patients with recurrent tumors after a long interval (more than 5 years), (2) 2 tumors from 1 patient with synchronous multiple squamous cell carcinoma in the contralateral side, and (3) 12 tumors from 6 patients with ambiguous histology but identical driver gene status. Synchronicity was marked based on the time intervals between resections, which yielded four synchronous (interval ≤ 6 months) and eight metachronous (interval > 6 months) tumor pairs. Clinical data, including sex, age, and smoking history, were extracted from the patients' medical records. The histology was assessed by experienced pathologists (Y.S.C. and H.S.S.) using CHA[25].

Genome sequencing

Genome sequencing was performed for the 12 patients using targeted sequencing (13 tumors from 6 patients) or WES (12 tumors from 6 patients). Targeted DNA sequencing was conducted using the TruSight Oncology 500 (TSO500; Illumina, San Diego, CA) [32] panel. The TSO500 panel comprises 523 cancer-related genes associated with potential SNVs and insertion-deletions (INDELs) and 59 genes associated with potential amplifications. DNA were extracted from 40 ng of formalin-fixed paraffin-embedded (FFPE) tissue using the Qiagen AllPrep DNA FFPE Kit (Qiagen, Hilden, Germany). Target enrichment was achieved through hybridization capture, and paired-end sequencing (2×150 bp) was conducted using a NextSeq550Dx

sequencer (Illumina) following the manufacturer's instructions.

For WES, DNA was extracted from FFPE tissue using the QIAamp DNA FFPE Tissue kit according to the manufacturer's protocols (Qiagen, Valencia, CA, USA). The concentration and quality of DNA were assessed using a NanoDrop spectrophotometer (Thermo Fisher Scientific, Waltham, MA, USA). WES was performed using the SureSelect Human All Exon V7 kit (Agilent Technologies, Santa Clara, CA, USA) and processed on the HiSeq 2500 platform to obtain a mean depth of 200× (Illumina). The raw sequencing reads were aligned to the GRCh38 genome reference using the BWA-MEM aligner (v0.7.17-r1188) [33]. Pre-processing steps, including MarkDuplicates, FixMateInformation, BaseRecalibrator, and ApplyBQSQ, were applied according to the GATK Best Practices (v4.2.0.0) [34].

Somatic mutation calling

For TSO500 sequencing, somatic mutations and CNVs called from the vendor provided bioinformatics pipeline were used. Variants with a VAF of <0.05 or >0.5 were filtered out. Germline filtering was performed using the population database within the TSO500 bioinformatics pipeline. All the somatic mutations were manually inspected by an experienced bioinformatics expert (J.W.) using the Integrative Genomics Viewer (IGV) (v2.8.6) [35]. CNV calls were further compared with histological diagnosis to confirm the sample match.

For WES, somatic mutations were detected using the GATK Mutect2 (v.4.2.0.0) tumor-only mode with the --f1r2-tar-gz argument to remove strand orientation bias artifacts. Cross-sample contamination was estimated with GetPileupSummaries and CalculateContamination included in GATK (v4.2.0.0). FFPE artifacts were further filtered out using SOBDetector (v1.0.2) [36] applied to the filtered Mutect2 output. To remove false calls and germline variants that satisfy any of the following criteria were filtered out: (i) VAF <0.05 and alternate allele count <5, (ii) VAF >0.5, (iii) overlap with germline calls from Strelka2 (v2.9.10) [37] and GATK HaplotypeCaller (v4.2.0.0) under default parameters, (iv) dbSNP (build 155) allele frequency >0.01, (v) gnomAD (v3.1) allele frequency >0.0001[38].

Tumor clonality analysis

Tumor clonality analysis was performed for in-house patients with disagreement between the original diagnosis and MeTel result. Bam files from WES were input into SuperFreq R package (v 1.4.5) [39] with default exome mode to generate river plots by the patients. The final classification was confirmed by comparing the clones containing tumors through the results of SuperFreq.

Supplementary Note

Supplementary Note 1

Of the 635 samples in the test sets, MeTel correctly classified IPM and MPLC in 623 cases, with an error rate of 1.89%, which outperformed the four other methods (error rate = 5.83%, 6.30%, 4.57%, and 11.97% in Mansuet-Lupo *et al.*, Chang *et al.*, Pei *et al.*, and Yang *et al.,* respectively). Notably, some test datasets overlapped with those used in for model development in previous algorithms. When these non-independent sets were excluded, the performance gap between MeTel and prior methods became even more pronounced. Excluding these non-independent sets revealed larger performance gaps in a robust comparison (error rate = 7.03%, 7.16%, 4.79%, and 12.79% in Mansuet-Lupo *et al.*, Chang *et al.*, Pei *et al.*, and Yang *et al.,* respectively).

To further challenge MeTel's robustness, we addressed the potential for labeling bias inherent in the composite test set, for which a 'gold standard' is unavailable. We performed a rigorous sensitivity analysis by constructing a high-confidence reference set. This set was composed exclusively of cases where the original, independent classifications—histologic and genomic—were concordant **(Table S12).** The agreement between these two distinct methodologies provides a stronger, more reliable ground truth for validation. On this stringent test set, MeTel demonstrated exceptional robustness by achieving perfect classification accuracy (100%), a kappa score of 1.00, and F1 scores of 1.00 for both IPM and MPLC **(Figure S6)**.

Supplementary Note 2

Patient 2 had three tumors, including one adenocarcinoma in the right middle lobe that was resected in 2009 and two synchronous adenocarcinomas in the right lower lobe that were resected in 2016. Upon the original histological evaluation, all tumors displayed an acinar-predominant pattern. However, the latter two tumors had a micropapillary component (5%–15%) that was not visible in the earlier tumor and exhibited a lepidic component (10%–30%), which can be interpreted as a noninvasive precursor lesion and can therefore be classified as MPLC. In contrast, MeTel predicted all tumors as "Confident" IPM. The patient was found to have a subdiaphragmatic metastasis on an abdominal CT two years after the last tumor resection. The clonality patterns of the three tumors, as determined by WES analysis, corroborated the diagnosis of IPM using MeTel. The final diagnosis of Patient 2 was readjusted to IPM, as predicted by MeTel.

Supplementary Note 3

Patient 6 was diagnosed with two synchronous squamous cell carcinomas located in the left lower and right upper lobes in 2015. At diagnosis, microscopic examination revealed that both tumors displayed similar histological features, including a keratinizing type, moderate differentiation, necrosis (10%–30%), and an inflammatory stroma, but there was no evidence of a precursor lesion, such as squamous dysplasia. Based on these findings, the tumors were originally classified as IPM on histological evaluation, whereas MeTel classified them as "Confident" MPLC. In the re-evaluation, clonality analysis using WES data revealed distinct clonal compositions in both tumors, which suggested that their true nature was MPLC. The patient has been under observation for 71.5 months without any recurrence. Based on this evidence, the final diagnosis was changed to MPLC.

Supplementary Note 4

Patient 7 had two adenocarcinomas detected at an interval of 8 years. The first tumor was identified in the left upper lobe in 2009 and exhibited a lepidic-predominant pattern (70%). One year later, brain metastasis was found, and the patient was treated with gamma knife surgery and navelbine chemotherapy. In 2017, a second tumor was discovered in the left lower

lobe, which also exhibited a lepidic-predominant pattern (90%). According to the histological criteria, the patient was initially diagnosed with MPLC because of the lepidic predominance in both tumors. In contrast, MeTel's prediction was "Confident" IPM. Re-analysis of the NGS panel revealed a similar CNV pattern. The patient received palliative care at another hospital without additional chemotherapy and was lost to follow-up 34 months after the last resection. Considering both the genomic similarity and the clinical course, the diagnosis of the patient was changed to IPM.

Supplementary Note 5

In Patient 11, lung adenocarcinoma first occurred in the right upper lobe in 2016, and another lung adenocarcinoma was found in the left lower lobe a year later, in 2017. The first tumor had an acinar-predominant (60%) pattern with minor solid (30%) and micropapillary (10%) components. The second tumor showed a much higher solid component (80%), thus resulting in MPLC diagnosis, because of to the difference in the predominant histologic type. In contrast, MeTel's call was "Confident" IPM. Reanalysis of the NGS data revealed similar chromosomal patterns, including copy number gains on chromosome 7 and a shared rare somatic variant (*AKT3* c.*5422T>A), which is indicative of IPM. Overall, these re-reviewed procedures for conflicting cases between histopathological diagnosis and MeTel demonstrate the clinical utility of MeTel for the diagnosis of patients with multifocal NSCLC.

Supplementary Note 6

Traditionally, time intervals between the primary and secondary tumors served as key indicators in distinguishing between IPM and MPLC; however, there was no clear threshold for this classification. Most NSCLC cases recur within two years of onset; hence, the two-year cutoff in the Martini criteria and the extended four-year cutoff by the American College of Clinical Pharmacy (ACCP) have been established based on such clinical incidence data. The model by Mansuet-Lupo *et al.* also utilizes a five-year cutoff for classifying MPLC. The lack of robustness in the use of time intervals was observed in five patients (Patients 1, 2, 5, 7, and 8) in our study; the patients had time intervals that exceeding five years but were diagnosed with IPM.

Our study also questioned the usefulness of histological patterns for classification. Irrespective of the type and organ of origin, precancerous lesions have consistently been employed as strong evidence of primary cancer rather than metastasis[40-42]. Similarly, in conventional histological criteria for lung cancer, the presence of an in situ component plays a crucial role in distinguishing primary cancer from metastasis[43,44]. In lung adenocarcinoma, the in situ component is characterized by neoplastic cells that are confined to the pre-existing alveolar wall without structural destruction[43,44]. This growth pattern is now termed "lepidic" and non-mucinous lung adenocarcinoma with a purely or predominantly lepidic pattern is classified as AIS, MIA, and lepidic adenocarcinoma, depending on the presence and size of invasive foci[44]. However, certain invasive adenocarcinomas demonstrate outgrowth along the alveolar wall, thus mimicking precancerous lesions[45]. In a recent study (as known as Delphi) [46], features for distinguishing in situ components from invasive patterns have been suggested, including extensive epithelial proliferation, desmoplasia, and altered alveolar structure, to rule out a considerable invasive pattern. MeTel's identification of two patients with lepidic patterns (Patients 2 and 7: see **Figure S7 and S8**), such as IPM, even without using any time or histological information, demonstrates the incompleteness of such clinical and histological features, which can be overridden by genome-based prediction. Thus, a lepidic pattern alone should not be considered definitive evidence for MPLC.

The ability to confirm the metastatic nature of squamous cell carcinoma in Patient 6 provides an encouraging perspective. Compared with lung adenocarcinoma, pulmonary squamous cell carcinoma exhibits a relatively lower frequency of driver gene alterations, such as *EGFR* mutations or *ALK* rearrangements[47]. Beyond the rarity of these specific gene alterations, the histological diversity of lung squamous cell carcinoma is less complex than that of lung adenocarcinoma and is divided into three categories: keratinizing, non-keratinizing, and basaloid[44]. When pulmonary squamous cell carcinoma presents as multiple intrapulmonary tumors, the lack of a significant driver gene and morphological heterogeneity makes it challenging to predict the tumor's clonal relationship[48]. In the histological assessment of multiple pulmonary squamous cell carcinomas, detailed morphological characteristics beyond the standard WHO classification were suggested to distinguish between IPM and MPLC, including degree of keratinization, necrosis, desmoplasia, and inflammation, and rare cytological features such as clear cell, papillary, basaloid, or sarcomatoid patterns[25]. However, in our study, the squamous cell carcinoma pair demonstrated indistinguishable cytological, structural, and stromal backgrounds, making it impossible to differentiate it histopathologically

from MPLC. The challenge of classification was similarly highlighted in a recent investigation focusing on squamous cell carcinoma using WES analysis[49]. Our study used WES to examine the clonal composition profile and confirmed that these tumors occurred independently. This finding was consistent with the outcome of the patients who remained recurrence-free for more than 5 years.

In Patient 11, the discrepancy in the predominant histologic components between the two tumors resulted in its classification as MPLC. However, a detailed histological review revealed that this was due to an increase in the high-grade (solid) component of a secondary tumor that appeared a year later. The International Association for the Study of Lung Cancer (IASLC) categorized this high-grade pattern as solid, micropapillary, and complex glandular in 2020 and established their correlation with a poor prognosis of non-mucinous adenocarcinoma[50,51]. Previous reports have documented an increased proportion of these high-grade components in metastatic cancers[14,52]. Through molecular analysis using MeTel, we rectified the histological discrepancy by confirming that the two tumors shared unique genomic variants. Consequently, variations in predominant histologic patterns should not be interpreted as MPLC but rather carefully assessed for potential progression during metastasis.

Supplementary Note 7

To assess the potential utility of integrating CNV into MeTel's probabilistic model, we explored a modified formulation of the α parameter used in likelihood estimation. In the current model, α is defined as 2 × VAF, which implicitly assumes diploidy and equal allele contribution. However, this assumption may not hold in the presence of CNVs, especially in tumors with copy number imbalance or loss of heterozygosity.

We proposed a CNV-adjusted α value defined as:

$$\alpha = total\_cn/major\_cn \times VAF$$

where:

- *total_cn* is the total copy number at the locus
- *major_cn* is the number of copies of the major allele
- *VAF* is the observed variant allele frequency in the tumor

This formulation assumes that the somatic mutation resides on the major allele. Under this assumption, the adjusted α reflects the expected frequency of a shared mutation given local CNV states.

A schematic example is shown in **Figure S9**, where both the unadjusted model and CNV-adjusted model yield the same α = 1/2 under common conditions (e.g., major_cn = 2, total_cn = 3, VAF = 1/3).

To evaluate the empirical impact of this adjustment, we applied the CNV-adjusted α formulation to our in-house dataset (n = 6) for which allele-specific CNV profiles were available. Classification results using the CNV-adjusted α values were then compared to those from the original model. The overall classification outcomes remained unchanged across all cases **(Figure S10 and Table S13)**.

These preliminary results support the theoretical feasibility of CNV-adjusted parameterization and motivate further investigation using phased or clonality-informed CNV data. While the current model remains robust, CNV integration may enhance interpretability in select edge cases or guide downstream biological interpretation.

Supplementary Note 8

We explored whether incorporating the distinct background prevalence of driver mutations across ethnicities could refine MeTel's performance. This hypothesis is supported by well-documented epidemiological differences, such as the frequencies of EGFR mutations (30%–51% in Asians vs. 7%–20% in non-Asians) and KRAS mutations (5%–10% in Asians vs. 18%–30% in non-Asians) [53-55].

To address this, we stratified NSCLC cases from the GENIE v15 cohort based on the "Primary Race" category into four groups: Asian (n=1,069), Black (n=1,018), Hispanic (n=663), and non-Asian (primarily White/Caucasian, n=13,837). The f_v values for all mutations were calculated separately for each group to construct the respective proof-of-concept models (e.g., MeTel-Asian, MeTel-Black).

For the external test datasets, which lacked explicit race annotations, ethnic background was inferred as a proxy based on the contributing institution's nationality (e.g., Korean or Chinese hospitals as Asian; French or U.S. hospitals as non-Asian). This proxy-based validation was

only applied to the broader Asian versus non-Asian comparison, as it was deemed insufficient for more granular validation of the Black- and Hispanic-specific models.

Our analysis, however, showed that this exploratory model did not lead to a consistent performance improvement over the general MeTel algorithm. In fact, its application in some cohorts resulted in a slight decrease in accuracy, indicating that the current Asian reference dataset is insufficient to build a statistically stable model, particularly for rarer variants. While applying a mismatched model (e.g., MeTel-non-Asian to the Asian subset) lowered accuracy, confirming the conceptual validity of the approach, the overall results underscore that its potential can only be realized with more substantial data.

Therefore, we present all population-specific models (Asian, Black, and Hispanic) not as definitive improvements, but as a framework provided to the community for future research. This work highlights a critical need for larger, more diverse, and well-annotated genomic reference databases to truly advance population-specific cancer diagnostics.

Supplementary Tables and Figures Overview

Table S1. Clinically Actionable Driver Alterations Used for Initial Discordance Filtering in MeTel

Table S2. Summary of test dataset

Table S3. Performance of algorithms. (635 cases from 20 test datasets)

Table S4. F1 scores for IPM and MPLC algorithms (635 cases from 20 test datasets)

Table S5. Patient characteristics of the targeted DNA sequencing (TSO500) and WES datasets

Table S6. Clinical and pathological characteristics of 25 multiple lung cancer tumors

Table S7. Results applied to in-house data

Table S8. CNV results (Pt7)

Table S9. CNV results (Pt11)

Table S10. Classification Performance of MeTel Across Different Values of Mutation Frequency Parameter (f_v)

Table S11. Classification results with and without VAF imputation

Table S12. Histology–genomic concordance across included test datasets.

Table S13. Classification results with and without CNV integration

Figure S1. Overview of the MeTel algorithm

Figure S2. Cases consistent between histologic predictions and MeTel analysis of the in-house dataset (WES).

Figure S3. Cases consistent between histologic predictions and MeTel analysis of the in-house dataset (TSO500 panel sequencing).

Figure S4. Sensitivity analysis of MeTel's classification outcomes across different values of the background mutation frequency parameter (f_v).

Figure S5. Sensitivity analysis for VAF imputation

Figure S6. Performance comparison in histology–genomic concordant cases

Figure S7. Pathologic slides of three tumors (T1, T2-1, and T2-2) of Patient 2

Figure S8. Pathological slides of two intrapulmonary tumors (T1 and T3) of Patient 7.

Figure S9. Conceptual model for CNV-integrated classification.

Figure S10. Comparison of MeTel classification scores with and without CNV integration

REFERENCE

1. National Comprehensive Cancer Network. NCCN Clinical Practice Guidelines in Oncology (NCCN Guidelines®): Non-small cell lung cancer (version 6.2025). Accessed 07/02, 2025. https://www.nccn.org/professionals/physician_gls/pdf/nscl.pdf

2. AACR Project GENIE Biopharma Collaborative Releases Colorectal Cancer Dataset. *Cancer Discov*. Jan 9 2023;13(1):OF6. doi:10.1158/2159-8290.CD-ND2022-0019

3. Milholland B, Dong X, Zhang L, Hao XX, Suh Y, Vijg J. Differences between germline and somatic mutation rates in humans and mice. *Nature Communications*. May 9 2017;8doi:ARTN 15183

10.1038/ncomms15183

4. Zheng R, Shen Q, Mardekian S, Solomides C, Wang ZX, Evans NR, 3rd. Molecular profiling of key driver genes improves staging accuracy in multifocal non-small cell lung cancer. *J Thorac Cardiovasc Surg*. Aug 2020;160(2):e71-e79. doi:10.1016/j.jtcvs.2019.11.126

5. Donfrancesco E, Yvorel V, Casteillo F, et al. Histopathological and molecular study for synchronous lung adenocarcinoma staging. *Virchows Arch*. Jun 2020;476(6):835-842. doi:10.1007/s00428-019-02736-0

6. Takahashi Y, Shien K, Tomida S, et al. Comparative mutational evaluation of multiple lung cancers by multiplex oncogene mutation analysis. *Cancer Sci*. Nov 2018;109(11):3634-3642. doi:10.1111/cas.13797

7. Qiu T, Li W, Zhang F, Wang B, Ying J. Major challenges in accurate mutation detection of multifocal lung adenocarcinoma by next-generation sequencing. *Cancer Biol Ther*. 2020;21(2):170-177. doi:10.1080/15384047.2019.1674070

8. Belardinilli F, Pernazza A, Mahdavian Y, et al. A multidisciplinary approach for the differential diagnosis between multiple primary lung adenocarcinomas and intrapulmonary metastases. *Pathol Res Pract*. Apr 2021;220:153387. doi:10.1016/j.prp.2021.153387

9. Mansuet-Lupo A, Barritault M, Alifano M, et al. Proposal for a Combined Histomolecular Algorithm to Distinguish Multiple Primary Adenocarcinomas from Intrapulmonary Metastasis in Patients with Multiple Lung Tumors. *J Thorac Oncol*. May 2019;14(5):844-856. doi:10.1016/j.jtho.2019.01.017

10. Bruehl FK, Doxtader EE, Cheng Y-W, Farkas DH, Farver C, Mukhopadhyay S. Does histological assessment accurately distinguish separate primary lung adenocarcinomas from intrapulmonary metastases? A study of paired resected lung nodules in 32 patients using a routine next-generation sequencing panel for driver mutations. *Journal of Clinical Pathology*. 2022;75(6):390-396. doi:10.1136/jclinpath-2021-207421

11. Roepman P, Ten Heuvel A, Scheidel KC, et al. Added Value of 50-Gene Panel Sequencing to Distinguish Multiple Primary Lung Cancers from Pulmonary Metastases: A Systematic Investigation. *J Mol Diagn*. Jul 2018;20(4):436-445. doi:10.1016/j.jmoldx.2018.02.007

12. Ezer N, Wang H, Corredor AG, et al. Integrating NGS-derived mutational profiling in the diagnosis of multiple lung adenocarcinomas. *Cancer Treat Res Commun*. 2021;29:100484. doi:10.1016/j.ctarc.2021.100484

13. Higuchi R, Nakagomi T, Goto T, et al. Identification of Clonality through Genomic Profile Analysis in Multiple Lung Cancers. *J Clin Med*. Feb 20 2020;9(2)doi:10.3390/jcm9020573

14. Chang JC, Alex D, Bott M, et al. Comprehensive Next-Generation Sequencing Unambiguously Distinguishes Separate Primary Lung Carcinomas From Intrapulmonary Metastases: Comparison with Standard Histopathologic Approach. *Clin Cancer Res*. Dec 1 2019;25(23):7113-7125. doi:10.1158/1078-0432.Ccr-19-1700

15. Duan J, Ge M, Peng J, et al. Application of large-scale targeted sequencing to distinguish multiple lung primary tumors from intrapulmonary metastases. *Sci Rep*. Nov 2 2020;10(1):18840. doi:10.1038/s41598-020-75935-4

16. Pei G, Li M, Min X, et al. Molecular Identification and Genetic Characterization of Early-Stage Multiple Primary Lung Cancer by Large-Panel Next-Generation Sequencing Analysis. *Front Oncol*. 2021;11:653988. doi:10.3389/fonc.2021.653988

17. Yang CY, Yeh YC, Wang LC, et al. Genomic Profiling With Large-Scale Next-Generation Sequencing Panels Distinguishes Separate Primary Lung Adenocarcinomas From Intrapulmonary Metastases. *Mod Pathol*. Mar 2023;36(3):100047. doi:10.1016/j.modpat.2022.100047

18. Shao J, Wang C, Li J, et al. A comprehensive algorithm to distinguish between MPLC and IPM in multiple lung tumors patients. *Ann Transl Med*. Sep 2020;8(18):1137. doi:10.21037/atm-20-5505

19. Haratake N, Toyokawa G, Takada K, et al. Programmed Death-Ligand 1 Expression and EGFR Mutations in Multifocal Lung Cancer. *Ann Thorac Surg*. Feb 2018;105(2):448-454. doi:10.1016/j.athoracsur.2017.09.025

20. Murphy SJ, Harris FR, Kosari F, et al. Using Genomics to Differentiate Multiple Primaries From Metastatic Lung Cancer. *J Thorac Oncol*. Sep 2019;14(9):1567-1582. doi:10.1016/j.jtho.2019.05.008

21. Chen X, Lu J, Wu Y, et al. Genetic features and application value of next generation sequencing in the diagnosis of synchronous multifocal lung adenocarcinoma. *Oncol Lett*. Sep 2020;20(3):2829-2839. doi:10.3892/ol.2020.11843

22. Suh YJ, Lee HJ, Sung P, et al. A Novel Algorithm to Differentiate Between Multiple Primary Lung Cancers and Intrapulmonary Metastasis in Multiple Lung Cancers With Multiple Pulmonary Sites of Involvement. *J Thorac Oncol*. Feb 2020;15(2):203-215. doi:10.1016/j.jtho.2019.09.221

23. Liu J, Mao G, Li Y, et al. Targeted deep sequencing helps distinguish independent primary tumors from intrapulmonary metastasis for lung cancer diagnosis. *J Cancer Res Clin Oncol*. Sep 2020;146(9):2359-2367. doi:10.1007/s00432-020-03227-5

24. Goto T, Hirotsu Y, Mochizuki H, et al. Mutational analysis of multiple lung cancers: Discrimination between primary and metastatic lung cancers by genomic profile. *Oncotarget*. May 9 2017;8(19):31133-31143. doi:10.18632/oncotarget.16096

25. Girard N, Deshpande C, Lau C, et al. Comprehensive histologic assessment helps to differentiate multiple lung primary nonsmall cell carcinomas from metastases. *Am J Surg Pathol*. Dec 2009;33(12):1752-64. doi:10.1097/PAS.0b013e3181b8cf03

26. Goodwin D, Rathi V, Conron M, Wright GM. Genomic and Clinical Significance of Multiple Primary Lung Cancers as Determined by Next-Generation Sequencing. *Journal of Thoracic Oncology*. 2021/07/01/ 2021;16(7):1166-1175. doi:https://doi.org/10.1016/j.jtho.2021.03.018

27. Patel SB, Kadi W, Walts AE, et al. Next-Generation Sequencing: A Novel Approach to Distinguish Multifocal Primary Lung Adenocarcinomas from Intrapulmonary Metastases. *J Mol Diagn*. Nov 2017;19(6):870-880. doi:10.1016/j.jmoldx.2017.07.006

28. Vignot S, Frampton GM, Soria J-C, et al. Next-Generation Sequencing Reveals High Concordance of Recurrent Somatic Alterations Between Primary Tumor and Metastases From Patients With Non–Small-Cell Lung Cancer. *Journal of Clinical Oncology*. 2013/06/10 2013;31(17):2167-2172. doi:10.1200/JCO.2012.47.7737

29. Yang SR, Chang JC, Leduc C, et al. Invasive Mucinous Adenocarcinomas With Spatially Separate Lung Lesions: Analysis of Clonal Relationship by Comparative Molecular Profiling. *J Thorac Oncol*. Jul 2021;16(7):1188-1199. doi:10.1016/j.jtho.2021.03.023

30. Tian H, Wang Y, Yang Z, et al. Genetic trajectory and clonal evolution of multiple primary lung cancer with lymph node metastasis. *Cancer Gene Ther*. Mar 2023;30(3):507-520. doi:10.1038/s41417-022-00572-0

31. Frankell AM, Dietzen M, Al Bakir M, et al. Author Correction: The evolution of lung cancer and impact of subclonal selection in TRACERx. *Nature*. Jul 2024;631(8022):E15. doi:10.1038/s41586-024-07738-w

32. Zhao C, Jiang T, Ju JH, et al. TruSight Oncology 500: Enabling Comprehensive Genomic Profiling and Biomarker Reporting with Targeted Sequencing. *bioRxiv*. 2020:2020.10.21.349100. doi:10.1101/2020.10.21.349100

33. Li H. Aligning sequence reads, clone sequences and assembly contigs with BWA-MEM. *arXiv: Genomics*. 2013;

34. Van der Auwera GA OCB. Genomics in the Cloud: Using Docker, GATK, and WDL in Terra. *O'Reilly Media*. 2020;

35. Robinson JT, Thorvaldsdottir H, Winckler W, et al. Integrative genomics viewer. *Nature Biotechnology*. Jan 2011;29(1):24-26. doi:10.1038/nbt.1754

36. Diossy M, Sztupinszki Z, Krzystanek M, et al. Strand Orientation Bias Detector to determine the probability of FFPE sequencing artifacts. *Briefings in Bioinformatics*. Nov 2021;22(6)doi:ARTN bbab186

10.1093/bib/bbab186

37. Kim S, Scheffler K, Halpern AL, et al. Strelka2: fast and accurate calling of germline and somatic variants. *Nature Methods*. Aug 2018;15(8):591-+. doi:10.1038/s41592-018-0051-x

38. Koboldt DC. Best practices for variant calling in clinical sequencing. *Genome Med*. Oct 26

2020;12(1):91. doi:10.1186/s13073-020-00791-w
39. Flensburg C, Sargeant T, Oshlack A, Majewski IJ. SuperFreq: Integrated mutation detection and clonal tracking in cancer. *Plos Computational Biology*. Feb 2020;16(2)doi:ARTN e1007603

10.1371/journal.pcbi.1007603
40. Lee AH. The histological diagnosis of metastases to the breast from extramammary malignancies. *J Clin Pathol*. Dec 2007;60(12):1333-41. doi:10.1136/jcp.2006.046078
41. Saida T, Tanaka YO, Matsumoto K, Satoh T, Yoshikawa H, Minami M. Revised FIGO staging system for cancer of the ovary, fallopian tube, and peritoneum: important implications for radiologists. *Japanese Journal of Radiology*. 2016/02/01 2016;34(2):117-124. doi:10.1007/s11604-015-0513-3
42. Jiang K, Al-Diffalha S, Centeno BA. Primary Liver Cancers—Part 1:Histopathology, Differential Diagnoses, and Risk Stratification. *Cancer Control*. 2018;25(1):1073274817744625. doi:10.1177/1073274817744625
43. Borczuk AC. Assessment of invasion in lung adenocarcinoma classification, including adenocarcinoma in situ and minimally invasive adenocarcinoma. *Modern Pathology*. 2012/01/01 2012;25(1):S1-S10. doi:10.1038/modpathol.2011.151
44. Board WCoTE. *WHO classification of tumours: Thoracic tumours*. World Health Organization (WHO); 2021:90-91.
45. Moore DA, Sereno M, Das M, et al. In situ growth in early lung adenocarcinoma may represent precursor growth or invasive clone outgrowth—a clinically relevant distinction. *Modern Pathology*. 2019/08/01 2019;32(8):1095-1105. doi:10.1038/s41379-019-0257-1
46. Thunnissen E, Beasley MB, Borczuk A, et al. Defining Morphologic Features of Invasion in Pulmonary Nonmucinous Adenocarcinoma With Lepidic Growth: A Proposal by the International Association for the Study of Lung Cancer Pathology Committee. *J Thorac Oncol*. Apr 2023;18(4):447-462. doi:10.1016/j.jtho.2022.11.026
47. Rekhtman N, Paik PK, Arcila ME, et al. Clarifying the spectrum of driver oncogene mutations in biomarker-verified squamous carcinoma of lung: lack of EGFR/KRAS and presence of PIK3CA/AKT1 mutations. *Clin Cancer Res*. Feb 15 2012;18(4):1167-76. doi:10.1158/1078-0432.Ccr-11-2109
48. Schneider F, Derrick V, Davison JM, Strollo D, Incharoen P, Dacic S. Morphological and molecular approach to synchronous non-small cell lung carcinomas: impact on staging. *Modern Pathology*. 2016/07/01 2016;29(7):735-742. doi:10.1038/modpathol.2016.66
49. Dacic S, Cao X, Bota-Rabassedas N, et al. Genomic Staging of Multifocal Lung Squamous Cell Carcinomas Is Independent of the Comprehensive Morphologic Assessment. *J Thorac Oncol*. Sep 16 2023;doi:10.1016/j.jtho.2023.09.275
50. Travis WD, Brambilla E, Noguchi M, et al. International Association for the Study of Lung Cancer/American Thoracic Society/European Respiratory Society International Multidisciplinary Classification of Lung Adenocarcinoma. *Journal of Thoracic Oncology*. 2011/02/01/ 2011;6(2):244-285. doi:https://doi.org/10.1097/JTO.0b013e318206a221
51. Woo W, Cha Y-J, Kim BJ, Moon DH, Lee S. Validation Study of New IASLC Histology Grading System in Stage I Non-Mucinous Adenocarcinoma Comparing With Minimally Invasive Adenocarcinoma. *Clinical Lung Cancer*. 2022/06/22/ 2022;doi:https://doi.org/10.1016/j.cllc.2022.06.004
52. Aokage K, Ishii G, Yoshida J, et al. Histological progression of small intrapulmonary metastatic tumor from primary lung adenocarcinoma. https://doi.org/10.1111/j.1440-1827.2010.02596.x. *Pathology International*. 2010/12/01 2010;60(12):765-773. doi:https://doi.org/10.1111/j.1440-1827.2010.02596.x
53. Liam CK, Pang YK, Poh ME. EGFR mutations in Asian patients with advanced lung adenocarcinoma. *J Thorac Oncol*. Sep 2014;9(9):e70-1. doi:10.1097/JTO.0000000000000251
54. Zhou W, Christiani DC. East meets West: ethnic differences in epidemiology and clinical behaviors of lung cancer between East Asians and Caucasians. *Chin J Cancer*. May 2011;30(5):287-92. doi:10.5732/cjc.011.10106
55. Gao B, Sun Y, Zhang J, et al. Spectrum of LKB1, EGFR, and KRAS mutations in chinese lung adenocarcinomas. *J Thorac Oncol*. Aug 2010;5(8):1130-5. doi:10.1097/JTO.0b013e3181e05016
